# Neo-actinomycins A and B, natural actinomycins bearing the 5*H*-oxazolo[4,5-*b*]phenoxazine chromophore, from the marine-derived *Streptomyces* sp. IMB094

**DOI:** 10.1038/s41598-017-03769-8

**Published:** 2017-06-15

**Authors:** Qiang Wang, Yixuan Zhang, Mian Wang, Yi Tan, Xinxin Hu, Hongwei He, Chunling Xiao, Xuefu You, Yiguang Wang, Maoluo Gan

**Affiliations:** Institute of Medicinal Biotechnology, Chinese Academy of Medical Sciences and Peking Union Medical College, Beijing, 100050 China

## Abstract

Neo-actinomycins A and B (1 and 2), two new natural actinomycins featuring an unprecedented tetracyclic 5*H*-oxazolo[4,5-*b*]phenoxazine chromophore, were isolated from the marine-derived actinomycete *Streptomyces* sp. IMB094. Their structures were elucidated by spectroscopic analyses. The presence of this ring system was proposed to originate from a condensation between actinomycin D (3) with α-ketoglutarate and pyruvate, respectively. Compound 1 showed potent cytotoxic activities against human cancer HCT116 and A549 cell lines in the nanomolar range (IC_50_: 38.7 and 65.8 nM, respectively) and moderate antibacterial activities against methicillin-resistant *Staphylococcus aureus* (MRSA) and vancomycin-resistant *Enterococci* (VRE) strains.

## Introduction

Actinomycins are well-known chromopeptides with potent cytotoxic and antibiotic activities, isolated from various *Actinomyces* strains^1^ and consisting of a tricyclic phenoxazinone chromophore attached to two pentapeptide lactone rings via amide bonds. More than 30 naturally occurring single congeners have been identified so far, and about 40 variants have been obtained by precursor-directed biosynthesis^2–4^. Natural actinomycins differ in amino acid composition of the peptidolactone side chains, whereas the chromophore (2-amino-4,6-dimethylphenoxazine-3-one-1,9-dicarboxylic acid, actinocin) is identical in all reported actinomycins^2^. Recently, *S. chrysomallus* and *S. parvulus* were reported to generate new C-demethylactinomycins lacking one or both methyl groups in their phenoxazinone chromophores when cultured with 3-hydroxyanthranilic acid^5^.

Actinomycin D is the most common actinomycin antibiotic and is used as an anticancer drug, particularly in the treatment of Wilms’ tumor and soft tissue sarcomas in children^6, 7^. Actinomycins intercalate DNA and inhibit DNA-primed RNA synthesis^8, 9^. As high toxicity of actinomycins restricts their clinical application, extensive structural redesign studies have been performed to improve their therapeutic index, leading to the synthesis of a number of analogs with structurally modified cyclopeptide rings or chromophore^1, 10^.

**Figure 1:**
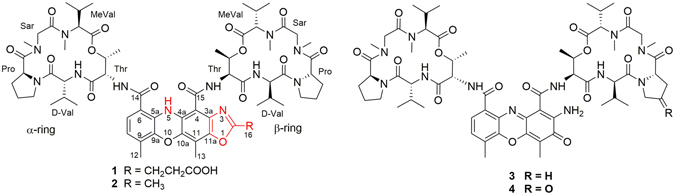
Chemical structures of compounds **1**–**4**.

In our ongoing research for new bioactive metabolites from marine-derived bacteria^11–13^, the crude extract of *Streptomyces* sp. IMB094 isolated from marine sediment showed potent antibacterial activity towards methicillin-resistant *Staphylococcus aureus* (MRSA) (MIC of <10 μg/mL) and cytotoxicity. Analysis of the exact by LC-UV-MS revealed metabolites with UV absorption similar to actinomycins^14, 15^. In addition to the observed UV absorption maximum at 443 nm which is characteristic for actinomycins^16^, the LC-UV-MS profile also showed a UV maximum at 410 nm for two of the metabolites (Supplementary Figure S1), which attracted our interest. Extensive investigation of the secondary metabolite composition of the IMB094 strain resulted in the isolation of a novel actinomycin chromophoric analog, neo-actinomycin A (**1**, Fig. 1), a new natural product, neo-actinomycin B (**2**), and two known actinomycins D and X_2_ (**3** and **4**). Structurally, the chromophore of neo-actinomycin A (**1**) contains a fourth oxazole ring fused with the actinocin moiety, forming a tetracyclic 5*H*-oxazolo[4,5-*b*]phenoxazine ring, which has never previously been found in naturally occurring actinomycins.

## Results and Discussion

Neo-actinomycin A (**1**) was isolated as a red powder, with molecular formula of C_66_H_90_N_12_O_18_ (HRESIMS), indicating 28 degrees of unsaturation. The IR spectrum of **1** suggested the presence of carboxyl (1743 cm^−1^), amide (1647 cm^−1^), and aromatic ring (1505 cm^−1^) functionalities. The ^1^H NMR spectrum of **1** (Table 1) in DMSO-*d*_6_ displayed characteristics of a typical peptide, with five NH protons (*δ*_H_ 7.2–11.7), 12 amino acid α-protons (*δ*_H_ 3.2–6.3), and two ester carbinol protons (*δ*_H_ 5.14 and 5.24). Furthermore, two *ortho*-coupled aromatic protons [*δ*_H_ 6.62 (d, H-8) and 7.11 (d, H-7)], four *N*Me singlets [*δ*_H_ 2.75 (s, 6H) and 3.19 (s, 6H)], as well as 12 additional methyl singlets or doublets (*δ*_H_ 0.7–2.3) were also observed in the ^1^H NMR spectrum. Analysis of the ^13^C NMR and DEPT spectra revealed the presence of 13 carbonyl signals (*δ*c 165–173) and 10 α-amino acid carbon signals (*δ*c 51–70), indicating that **1** was a peptide. Additional carbon NMR data showed the presence of 16 methyl groups, 8 methylenes, 2 sp^2^ methines, 6 sp^3^ methines, and 11 sp^2^ quaternary carbons (*δ*c 100–165), in agreement with spectral features observed for actinomycins^2, 15^. Comparison of NMR data of **1** with actinomycin D (Tables S1 and S2) revealed similar resonances in the two peptidolactone rings and differences in the chromophore moiety, indicating that the chromophore moiety of **1** was significantly modified. This conclusion was corroborated by the UV spectrum, which showed a bathochromic shift (absorption maximum at 410 nm) compared to actinomycin D.

**Table 1:** NMR Data for Neo-actinomycin A (**1**) in DMSO-*d*_6_^a^_._^a1^H and ^13^C NMR data were recorded at 600 and 150 MHz, respectively.

no.	pentapeptidolactone (*α*-ring)		no.	pentapeptidolactone (*β*-ring)	
	*δ*_H_, m (*J* in Hz)	*δ*_C,_ type		*δ*_H_, m (*J* in Hz)	*δ*_C,_ type
Thr 1		169.2, C	Thr 1		169.4, C
2	4.97, dd (9.0, 2.4)	53.9, CH	2	4.84, dd (8.4, 2.4)	54.9, CH
3	5.14, qd (6.0, 2.4)	72.3, CH	3	5.24, qd (6.0, 2.4)	71.9, CH
4	1.17, d (6.0)	16.3, CH_3_	4	1.20, d (6.0)	16.7, CH_3_
NH	7.25, d (9.0)		NH	9.66, d (8.4)	
Val 1		170.6, C	Val 1		170.7, C
2	3.50, m	57.4, CH	2	3.49, m	57.4, CH
3	1.88, m	30.1, CH	3	1.88, m	30.2, CH
4	0.92, d (6.6)	18.6, CH_3_	4	0.94, d (6.6)	18.6, CH_3_
5	0.69, d (7.2)	19.1, CH_3_	5	0.70, d (7.2)	19.1, CH_3_
NH	8.42, d (6.0)		NH	8.29, d (5.4)	
Pro 1		173.0, C	Pro 1		173.0, C
2	6.24, dd (9.0,3.0)	54.8, CH	2	6.21, dd (9.0,3.0)	54.8, CH
3	2.10, m; 1.74, m	31.1, CH_2_	3	2.10, m; 1.74, m	31.2, CH_2_
4	1.91, m; 1.67, m	22.7, CH_2_	4	1.91, m; 1.67, m	22.8, CH_2_
5	3.49, m; 3.27, m	46.4, CH_2_	5	3.50, m; 3.27, m	46.4, CH_2_
Sar 1		167.0, C	Sar 1		167.1, C
2	4.80, d (18.0); 4.08, d (18.0)	51.2, CH_2_	2	4.78, d (18.0); 4.08, d (18.0)	51.2, CH_2_
NMe	2.75, s	34.4, CH_3_	NMe	2.75, s	34.4, CH_3_
MeVal 1		168.1, C	MeVal 1		168.2, C
2	3.23, d (9.6)	69.5, CH	2	3.23, d (9.6)	69.6, CH
3	2.54, m	26.5, CH	3	2.54, m	26.5, CH
4	0.97, d (6.6)	21.0, CH_3_	4	0.98, d (6.6)	21.1, CH_3_
5	0.79, d (6.6)	18.8, CH_3_	5	0.80, d (6.6)	18.8, CH_3_
NMe	3.19, s	38.6, CH_3_	NMe	3.19, s	38.8, CH_3_
chromophore					
2		165.8, C	10^a^		139.5, C
3^a^		132.9, C	11		111.8, C
4		100.2, C	11^a^		143.5, C
4^a^		132.0, C	12	2.13, s	14.8, CH_3_
5	11.74, s		13	2.27, s	8.9, CH_3_
5^a^		131.3, C	14		166.6, C
6		113.4, C	15		165.4, C
7	7.11, d (8.4)	122.0, CH	16	3.17, m	23.3, CH_2_
8	6.62, d (8.4)	122.0, CH	17	2.89, m	30.3, CH_2_
9		127.5, C	18		173.1, C
9^a^		140.7, C			

**Figure 2:**
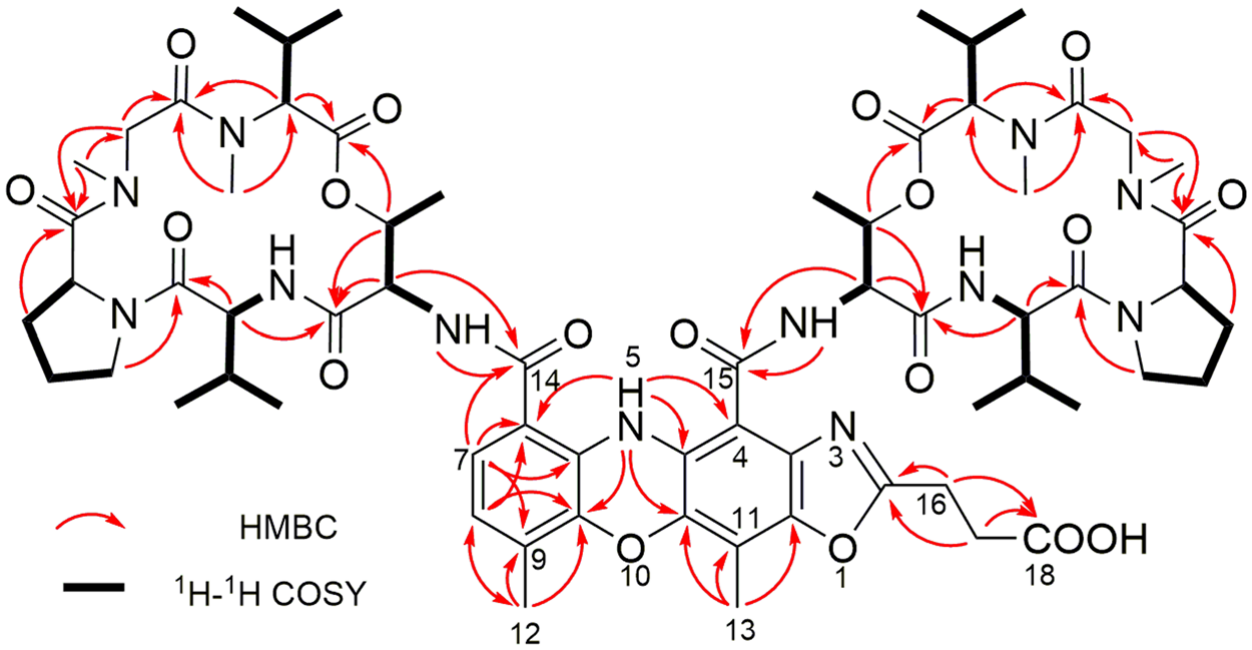
Key HMBC and ^1^H-^1^H COSY correlations of **1**.

Extensive analysis of 2D NMR (HSQC, COSY, and HMBC) spectroscopic data revealed 10 amino acid residues in **1**: 2 × Thr, 2 × Val, 2 × Pro, 2 × sarcosin (Sar), 2 × MeVal. Amino acid sequences in the two peptidolactone units in **1** were determined to be identical with those of actinomycin D using HMBC correlations (Fig. 2). The chromophore moiety of **1** was established as follows. The *ortho*-coupled aromatic protons H-7 and H-8 correlated with two overlapped carbons at *δ*_C_ 122.0 (C-7 and C-8) in the HSQC spectrum. In the HMBC spectrum, H-8 correlated with C-6 (*δ*_C_ 113.4), C-9a (*δ*_C_ 140.7), and the methyl carbon C-12 (*δ*_C_ 14.8), whereas H-7 showed cross-peaks with C-5a (*δ*_C_ 131.3), C-6, C-9 (*δ*_C_ 127.5), and the carboxyl carbon C-14 (*δ*_C_ 166.6). Further, the exchangeable NH proton at *δ*_H_ 11.74 correlated with C-4 (*δ*_C_ 100.2), C-4a, C-6, C-9a, and C-10a (*δ*_C_ 139.5), whereas the methyl protons at *δ*_H_ 2.27 (s, H_3_–13) showed correlation with C-10a, C-11 (*δ*_C_ 111.8), and C-11a. These HMBC correlations, combined with the chemical shift of the carbon resonances, indicated the presence of the 9,11-dimethyl-5*H*-phenoxazine moiety. The chemical shift of C-11a (*δ*_C_ 143.5) suggested that this carbon participated in a C-O single bond in **1**, instead of the C=O double bond found in conventional actinomycins. Carbon resonance at *δ*_C_ 173.1 (C-18) indicated the presence of a free carboxylic acid group. HMBC correlation of the mutually coupled methylene protons H_2_-16 (*δ*_H_ 3.17) and H_2_-17 (*δ*_H_ 2.89) with C-18 and the remaining downfield quaternary carbon at *δ*_C_ 165.8 (C-2) revealed the presence of N=CCH_2_CH_2_COOH substructure moiety. This structure fragment, together with two peptidolactone units and the 11a-oxy-9,11-dimethyl-5*H*-phenoxazine moiety, accounted for 27 degrees of unsaturation. To fulfill 28 degrees of unsaturation, an additional ring in **1** was required, indicating that the quaternary carbon C-2 was connected with C-3 and C-11a through a nitrogen and an oxygen atom, respectively, forming an oxazole ring and completing the backbone of **1**.

The absolute configurations of amino acids were determined using advanced Marfey’s method after acid hydrolysis^17, 18^. Comparison of retention times between l- and d/l-FDLA (1-fluoro-2,4-dinitrophenyl-5-leucine amide) adducts (Supplementary Figure S2) assigned l configurations for Thr, MeVal, and Pro residues and d configuration for Val. With its structure fully resolved, **1** was established as a new member of the actinomycin family and named neo-actinomycin A.

The molecular formula of neo-actinomycin B (**2**) was elucidated as C_64_H_88_N_12_O_16_ by HRESIMS, with one C_2_H_2_O_2_ unit less than **1**. The UV and NMR data (Supplementary Tables S3 and S4) of **2** were similar to those of **1**. The major difference between the NMR data of **1** and **2** was the absence of signals corresponding to the carboxyethyl group in **2** that were present in **1**. A new methyl signal was observed at *δ*_H_/*δ*_C_ 2.63/14.4 in **2**, suggesting that the carboxyethyl group in **1** was replaced by methyl in **2**, which was confirmed by 2D NMR data analysis (Supplementary Figure S3). This is the first report on naturally occurring compound **2**, which was previously synthesized as a derivative of actinomycin D^14^. It should be noted that prior to this study, no NMR data have been reported for this compound. Compound **2** was assigned the trivial name neo-actinomycin B since it had not been described from nature previously.

**Figure 3:**
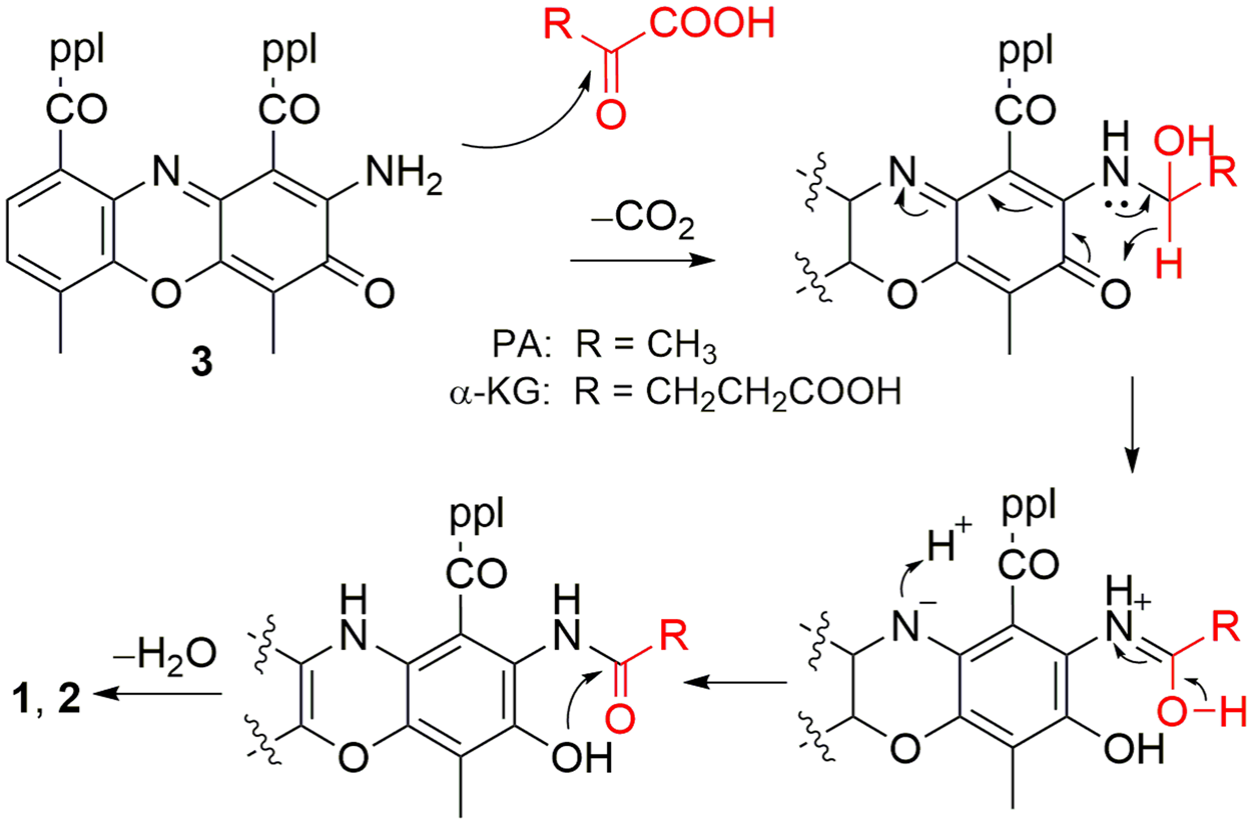
Plausible biosynthesis pathway for neo-actinomycin A (**1**).

A plausible biosynthetic pathway (Fig. 3) for neo-actinomycins A and B (**1** and **2**) is proposed, starting from actinomycin D (**3**) and the tricarboxylic acid (TCA) cycle intermediates^19^, α-ketoglutarate (α-KG) and pyruvate (PA), respectively. Nucleophilic addition of the free 2-amino group of **3** to the α-keto function of α-ketoglutarate (or pyruvate), followed by a cyclization, would give rise to **1** (or **2**). The cyclization process might proceed by a concerted intramolecular hydrogen shift mechanism as proposed previously^20, 21^. To test the biosynthetic hypothesis we investigated the possibility of precursor-directed *in situ* synthesis of **1** and **2** by adding the proposed precursors α-ketoglutaric acid and pyruvic acid (1 mg/mL) after cultivation of *Streptomyces* sp. IMB094. LC-MS analysis indicated 12-fold increase in the production of **1** in α-ketoglutaric acid-supplemented cultures compared to unsupplemented control (Figure S4). It is interesting to note that the yield of **1** and **2** both increased about 6-fold 24 h after pyruvic acid was added into the cultures. A possible explanation is that the exogenous pyruvic acid is converted into α-ketoglutaric acid through the *in vivo* tricarboxylic acid (TCA) cycle biosynthesis pathway during cultivation, but this remains to be demonstrated.

We further explored the possibility of *in vitro* transformation of the precursors in a variety of solvents, including the fermentation M8 media, H_2_O, and MeOH (Figures S5 and S6). After incubation of **3** and α-ketoglutaric acid at 28 °C for 36 h, we observed approximately 10% conversion of **3** to **1** in H_2_O and in M8 media, but no production of **1** in MeOH (Figure S5). Incubation with pyruvic acid under the same conditions lead to about 50% conversion of **3** to **2** in H_2_O and M8 media, and only 5% conversion in MeOH (Figure S6). Further investigation revealed that the conversion rates in MeOH and H_2_O varied slightly under pH 1.0 and 2.0 conditions, but dramatically decreased under pH 4.0 (Table S6). The low conversion rate in MeOH could be explained by the strong hydrogen bond between the 2-amino group and the pentapetidolactone^14, 22^. These results suggest that **1** and **2** were formed by a condensation of actinomycin D with α-ketoglutarate and pyruvate, respectively.

In a preliminary investigation of the biosynthetic origin of α-ketoglutarate and pyruvate, strain IMB094 was cultured in parallel in the M8 medium and in the chemically defined medium, the galactose-glutamate-mineral salts (GGM) medium, which was proved to be good for actinomycin production for *S. antibioticus* 3720^23^. After cultivation at 28 °C for six days, the marine-derived strain IMB094 grew considerably better in the M8 medium containing 3.0% artificial sea salts than in the GGM media. LC-MS analysis of the metabolites showed that **1** and **2** were not present in the GGM fermentation broth. Compounds **3** and **4** were produced in the cultures grown in GGM medium; but their yield rates were only 7% of those in the M8 medium (Figure S7 and Table S7). Although α-ketoglutarate and pyruvate could not be detected in both media prior to cultivation by LC-MS analysis, they were found to be present in the extracts of both cultures grown in the M8 and GGM media (Figure S8 and Table S7). This suggested that α-ketoglutarate and pyruvate were formed during cultivation. Since galactose and l-glutamic acid were the only two organics in the GGM medium, the presence of pyruvate in the cultures (21.4 μg/mL, even higher than in the M8 cultures) indicated that α-ketoglutarate and pyruvate in the cultures did not directly derive from the exogenous alanine and glutamate in the medium by deamination. A plausible origin for the two precursors was from the TCA cycle biosynthesis pathway of the strain. Further biosynthesis studies of neo-actinomycins A and B were being undertaken.

Compounds **1** and **2** were evaluated for their *in vitro* antibacterial activity against a series of Gram-positive and Gram-negative drug-resistant pathogens (Table 2) and for cytotoxicity against two human cancer cell lines (Table 3) using actinomycin D (**3**) as a control. Compound **1** showed moderate antibacterial activities against methicillin-resistant *Staphylococcus aureus* (MRSA) and vancomycin-resistant *Enterococci* (VRE) strains with MIC values of 16–64 μg/mL, 64–256-fold less active than **3**. No antibacterial effects were observed for **2** (MIC > 128 μg/mL). Neo-actinomycin A (**1**) exhibited potent cytotoxicities against HCT116 and A549 cancer cell lines at nanomolar concentrations (IC_50_: 38.7 and 65.8 nM), approximately 800-fold decrease in activity relative to **3**. This could be explained by the loss of planarity of the chromophore and the 2-amino group, which interacts with the DNA backbone by forming an additional hydrogen bond with cytosine residues^9, 24^. Compound **2** differs from **1** only in the substituent at C-2 of the oxazole ring (methyl vs. carboxyethyl), which results in a significant 10-fold decrease in activity. A possible reason for the higher potency of **1** than **2** might be that the carboxyl group in **1** forms an additional interaction with the DNA as the 2-amino group in **3** ^24^.

**Table 2:** Antimicrobial Bioassay Results (MIC, μg/mL) for compounds **1**–**4**. ^a^Methicillin-susceptible *Staphylococcus aureus*. ^b^Methicillin-resistant *S. aureus*. ^c^Methicillin-susceptible *S*. *epidermidis*. ^d^Methicillin-resistant *S*. *epidermidis*. ^e^Vancomycin-susceptible *Enterococcus*. ^f^Vancomycin-resistant *Enterococcus*. ^g^Extended-spectrum beta-lactamase-producing strain. ^h^New Delhi metallo-beta-lactamase 1.

microorganism	strain no.	phenotype	1	2	3	4	levofloxacin
*Staphylococcus aureus*	ATCC 29213	MSSA^a^	16	>128	0.25	0.25	0.125
*Staphylococcus aureus*	15	MSSA	32	>128	0.25	0.125	0.125
*Staphylococcus aureus*	13–17	MSSA	32	>128	0.25	0.125	0.125
*Staphylococcus aureus*	ATCC 33591	MRSA^b^	16	>128	0.25	0.25	0.125
*Staphylococcus aureus*	13–18	MRSA	64	>128	0.25	0.25	32
*Staphylococcus epidermidis*	ATCC 12228	MSSE^c^	64	>128	0.5	0.25	0.125
*Staphylococcus epidermidis*	13–1	MSSE	64	>128	0.5	0.125	0.25
*Staphylococcus epidermidis*	13–3	MRSE^d^	32	>128	0.5	0.25	64
*Enterococcus faecalis*	ATCC 29212	VSE^e^	16	>128	0.25	0.125	1
*Enterococcus faecalis*	13–4	VSE	16	>128	0.25	0.06	1
*Enterococcus faecalis*	ATCC 51299	VRE^f^	16	>128	0.25	0.125	1
*Enterococcus faecalis*	ATCC 51575	VRE	16	>128	0.25	0.25	1
*Enterococcus faecium*	ATCC 700221	VRE	32	>128	0.125	0.125	64
*Enterococcus faecium*	13–7	VSE	32	>128	0.25	0.25	64
*Enterococcus faecium*	12–1	VRE	32	>128	0.25	0.25	64
*Escherichia coli*	ATCC 25922		>128	>128	64	128	≤0.03
*Escherichia coli*	1515		>128	>128	64	128	≤0.03
*Escherichia coli*	14–10		>128	>128	32	128	2
*Escherichia coli*	14–11	ESBLs^g^	>128	>128	64	64	16
*Klebsiella pneumoniae*	ATCC 700603	ESBLs	>128	>128	>128	>128	0.5
*Klebsiella pneumoniae*	7		>128	>128	>128	>128	0.06
*Klebsiella pneumoniae*	ATCC BAA-2146	NDM-1^h^	>128	>128	128	128	>128
*Klebsiella pneumoniae*	14–4		>128	>128	>128	>128	0.125
*Klebsiella pneumoniae*	14–15	ESBLs	>128	>128	>128	>128	0.25
*Pseudomonas aeruginosa*	ATCC 27853		>128	>128	>128	>128	1
*Pseudomonas aeruginosa*	PAO1		128	>128	>128	>128	4
*Pseudomonas aeruginosa*	13–46		>128	>128	>128	>128	0.25
*Acinetobacter calcoacetious*	ATCC 19606		>128	>128	32	32	0.125
*Enterobacter cloacae*	ATCC 43560		>128	>128	32	16	≤0.03
*Enterobacter aerogenes*	ATCC 13048		>128	>128	>128	>128	0.125
*Serratia marcescens*	ATCC 21074		>128	>128	>128	>128	0.125
*Morganella morganii*	ATCC 25830		>128	>128	128	>128	0.125
*Providentia rettgeri*	ATCC 31052		>128	>128	>128	>128	≤0.03
*Proteus vulgaris*	ATCC 29905		>128	>128	>128	>128	≤0.03
*Proteus mirabilis*	13–1		>128	>128	>128	128	0.125
*Stenotrophomonas maltophilia*	ATCC 13636		>128	>128	32	16	1
*Citrobacter freundii*	ATCC 43864		>128	>128	>128	>128	0.125

**Table 3:** Cytotoxicity of Compounds **1**–**4**.

Compound	IC_50_ (nM)	
	A549	HCT116
**1**	65.8	38.7
**2**	952.3	339.1
**3**	0.095	0.045
**4**	0.0025	0.0075

## Conclusion

In summary, we have isolated two new natural actinomycins, neo-actinomycins A and B (**1** and **2**) from a marine-derived *Streptomyces* sp. strain. Neo-actinomycin A represents the first reported instance of natural actinomycins possessing an unusual 5*H*-oxazolo[4,5-*b*]phenoxazine ring system. Investigation of biosynthesis indicated that neo-actinomycins A and B are formed by a condensation of α-ketoglutarate and pyruvate, with the actinocin chromophore of actinomycin D.

## Methods

### General Experimental Procedures

Optical rotations were determined using a Perkin-Elmer model 343 polarimeter. UV and CD spectra were recorded on an Applied Photophysics Chirascan spectropolarimeter. IR spectra were recorded on a Nicolet 5700 FT-IR microscope spectrometer (FT-IR microscope transmission). 1D- and 2D-NMR spectra were obtained at 600 MHz for ^1^H and 150 MHz for ^13^C, respectively, on a Bruker AVANCE III HD 600 MHz spectrometers in DMSO-*d*_6_ (*δ*_H_ 2.500 and *δ*_C_ 39.520), and MeOH-*d*_4_ (*δ*_H_ 3.310 and *δ*_C_ 49.000) with solvent peaks used as references. HRESIMS data were obtained using a Thermo LTQ Orbitrap XL mass spectrometer. LC-MS analysis was performed using an Agilent 1290 Infinity II LC coupled with a 1100 LC/MSD Model G1946D mass spectrometer. Preparative HPLC was conducted on a Shimadzu LC-20AP pump with a SPD-M20A photodiode array detector. TLC was carried out with glass precoated silica gel GF254 plates. Spots were visualized under UV light or by spraying with 7% H_2_SO_4_ in 95% aqueous EtOH followed by heating.

### Producing Microorganism and Fermentation

The producing microorganism, *Streptomyces* sp. IMB094, was isolated from a marine sediment sample collected at a depth of ca. 40 m from Heishijiao Bay, Dalian, China. On the basis of the 16S rDNA gene sequence (GenBank accession no. KY111376) analysis, strain IMB094 is most closely related to *Streptomyces antibioticus* NBRC 12838 (GenBank no. AB184184). Strain IMB094 was grown on ISP4 agar plates prepared with 3.0% artificial sea salt at 28 °C for 10 days and then inoculated into 300 replicate 500 mL Fernbach flasks each containing 100 mL of sterile M8 medium (composed of 10 g of starch, 25 g of glucose, 10 g of cottonseed flour, 3 g of peptone, 5 g of CaCO_3_, 0.1 g of KH_2_PO_4_, 0.1 g of MgSO_4_, and 30 g of artificial sea salt in 1L of H_2_O) and cultured on rotary shakers (200 rpm) at 28 °C for 5 days.

### Isolation

The culture broth (30L) was separated into the mycelia and the supernatant by centrifugation. The mycelial cake was extracted four times with acetone. The supernatant was subjected to an XAD-7HP macroporous adsorbent resin column (6L). The column was washed with H_2_O, and eluted successively with 50% and 90% aqueous acetone. The two latter fractions were combined with the mycelial acetone extracts and then concentrated under reduced pressure to afford a crude extract (25 g). The extract was applied to silica gel column chromatography (CC) eluting with a step-gradient of CH_2_Cl_2_−MeOH (50:1–0:100, v/v) to give 10 fractions (F_1_–F_10_) on the basis of TLC results. The fractions F_5_ (20:1, 0.36 g), F_6_ (9:1, 0.54 g), and F_7_ (9:1, 0.65 g) were separately applied to Sephadex LH-20 CC eluting with 90% aqueous MeOH and then separated on a preparative reversed-phase (RP) C_18_ HPLC column (Capcell MGII 5 μm, 20 mm × 250 mm, 10 mL/min) using a linear gradient of 75–100% aqueous MeOH over 60 min to yield impure neo-actinomycins A and B as major components (retention times: A, 23.96–30.60 min; B, 34.25–41.26 min). Final purification were achieved by repeated RP-HPLC on a 10 mm × 250 mm C-18 column (Nacalai Cosmosil MGII) using 58% MeCN in 0.5% formic acid for **1** and 75% MeCN in 0.5% formic acid for **2** as a mobile phase (4 mL/min flow rate, 400 nm detection) to obtain 28 mg of **1** (*t*_R_: 28.41 min) and 23 mg of **2** (*t*_R_: 15.42 min). Typical recoveries of compounds **1**–**4** from 1 L cultures were 0.9, 0.8, 23, 1.8 mg, respectively.

#### Neo-actinomycin A (**1**)

Red amorphous powder; [α]^20^_D_ –97.7 (*c* 0.35, MeOH); UV (MeOH) *λ*_max_ (log*ε*) 251 (4.50), 410 (4.12) nm; ECD (*c* 1.89 × 10^−4^ M, MeOH) *λ*_max_ (Δ*ε*) 210 (−27.4), 242 (+1.1), 277 (−7.0), 405 (−2.0) nm; IR *v*_max_ 3397, 2922, 1743, 1647, 1505, 1467, 1259, 1194, 1098 cm^−1^; ^1^H NMR (DMSO-*d*_6_, 600 MHz) and ^13^C NMR (DMSO-*d*_6_, 150 MHz) data, Table 1; HRESIMS *m/z* 1339.6542 [M+H]^+^ (calcd for C_66_H_91_N_12_O_18_, 1339.6569), 1361.6355 [M+Na]^+^ (calcd for C_66_H_90_N_12_O_18_Na, 1361.6388).

#### Neo-actinomycin B (**2**)

Red amorphous powder; [α]^20^_D_ −105.2 (*c* 0.28, MeOH); UV (MeOH) *λ*_max_ (log*ε*) 251 (4.57), 409 (4.13) nm; ECD (*c* 1.95 × 10^−4^ M, MeOH) *λ*_max_ (Δ*ε*) 210 (−43.4), 242 (+1.1), 277 (−10.4), 403 (−3.0) nm; IR *v*_max_ 3394, 2922, 1745, 1647, 1504, 1467, 1255, 1193, 1126, 1098 cm^−1^; ^1^H NMR (DMSO-*d*_6_ and CD_3_OD, 600 MHz) data, Supplementary Table S4; ^13^C NMR (DMSO-*d*_6_ and CD_3_OD, 150 MHz) data, Supplementary Table S3; HRESIMS *m/z* 1298.6775 [M+NH_4_]^+^ (calcd for C_64_H_92_N_13_O_16_, 1298.6779), 1303.6327 [M+Na]^+^ (calcd for C_64_H_88_N_12_O_16_Na, 1303.6333).

### Marfey’s Analysis of Compound 1

Compound **1** (0.9 mg) was dissolved in 0.5 mL of 6 M HCl and heated to 110 °C for 16 and then the hydrolysate was evaporated to dryness, redissolved in H_2_O (120 *μ*L) and divided into two portions. To each portion (60 *μ*L) was added 100 *μ*L of a 1% solution of 1-fluoro-2,4-dinitrophenyl-5-l-alanine amide (l-FDAA) or 1% d-FDAA in acetone and 20 *μ*L of 1 M NaHCO_3_. The reaction mixture was heated at 40 °C for 1 h, cooled to room temperature, neutralized with 2 M HCl (10 *μ*L), and diluted with MeCN (100 *μ*L). The analyses of l- and l/d-FDLA derivatives were carried out with ESI-LC/MS using the following conditions: column, Cosmosil MG II C_18_ column (5 μm, 4.6 × 150 mm); flow rate, 1.0 mL/min; solvent A, 0.1% formic acid; solvent B, MeCN; linear gradient elution from 25–55% of B in A over 60 minutes; UV detection at 340 nm; column temperature, 40 °C.

The retention times of the l-FDLA derivatives for compound **1** were as follows: d-Val (44.35 min), l-Thr (17.79 min), l-Pro (24.75 min), and l-MeVal (37.35 min). The retention times of the l/d-FDLA derivatives for compound **1** were as follows: d-Val (44.29, 32.04 min), l-Thr (17.75, 25.87 min), l-Pro (24.78, 30.07 min), and l-MeVal (37.31, 44.80 min). These results established the d-configuration for Val and l-configurations for other amino acid residues.

### Cytotoxicity Assay

The cytotoxicities of the tested compounds against the human cancer cells A549 (lung adenocarcinoma) and HCT116 (colon carcinoma) were evaluated by the sulforhodamine B (SRB) assay using a standard protocol developed by the NCI^25^. A549 and HCT116 cells were maintained in dulbecco’s modified eagle medium(DMEM, Hyclone) and RPMI 1640 medium (Hyclone), respectively. All media contained 100 units/mL of penicillin, 100 mg/mL of streptomycin and 10% fetal bovine serum. For the cytotoxicity assays, cells were inoculated into 96-well plates at a concentration of 4000 cells per well. After incubation at 37 °C under a humidified atmosphere containing 5% CO_2_ for 24 h, cells were treated with various concentrations of test compounds in triplicate and further incubated for 48 h. Cell proliferation was determined by the SRB assay. The IC_50_ value was defined as the compound concentration which produces 50% inhibition of cell growth during 2 days of compound treatment and calculated using GraphPad Prism 5 software with nonlinear regression fit analysis.

### Procedure for precursor-directed *in situ* synthesis

Spore suspensions of *Streptomyces* sp. IMB094 were inoculated into 100 mL of M8 medium in 500 mL flasks. After incubation at 28 °C for 4 days, the cultures (each 30 mL) were transferred to two new 100 mL flasks and then α-ketoglutaric and pyruvic acids were separately added at a final concentration of 1 mg/mL. The remaining cultures (30 mL) without supplemental precursors were grown in parallel as negative control. The cultures were further grown at 28 °C for 1 day at 200 rpm. 30 mL samples of culture broth were concentrated in vacuum to dryness. The mixtures were then extracted with 30 mL of MeOH in an ultrasonic bath at 40 °C for 30 minutes. The mixture was spun down and the clear methanol extract was evaporated to dryness and redissolved in 300 μL of MeOH-EtOAc (5:1) solvents. 5 μL of the extract was analyzed by HPLC-ESI-MS (Capcell MGII C-18 5 μM, 4.6 mm × 150 mm; mobile phase A: 5 mM ammonium formate aqueous solution; B: MeCN; gradient conditions: 0–20 min, linear gradient 40–80% B; 25 min, 80% B; flow rate: 1 mL/min). If not mentioned otherwise, LC-MS analysis in this study was performed using the conditions described above.

### Comparison analysis of the metabolites in M8 and GGM media

Spores of strain IMB094 were inoculated into 500 mL Erlenmeyer flasks containing 100 mL of TCG medium (3 g of tryptone, 5 g of casitone, 4 g of glucose, and 30 g of artificial sea salt in 1 L of H_2_O)^26^. The cultures were grown at 28 °C for 3 days. 5 mL of seed medium was inoculated to 500 mL Erlenmeyer flasks containing 100 mL of the M8 and GGM (10 g of galactose, 2 g of l-glutamic acid, 1 g of K_2_HPO_4_, 0.025 g of MgSO_4_·7H_2_O, 0.025 of ZnSO_4_·7H_2_O, 0.025 of FeSO_4_·7H_2_O, 0.025 of CaCl_2_·2H_2_O in 1 L of H_2_O, pH 7.2) media in 500 mL Erlenmeyer flasks. Fermentation was carried out 28 °C for 6 days with agitation at 180 rpm. 50 mL samples of culture broth were concentrated in vacuum to dryness. The mixtures were then extracted with 50 mL of MeOH in an ultrasonic bath at 40 °C for 30 minutes. The mixture was spun down and the clear methanol extract was evaporated to dryness and redissolved in 1200 μL of MeOH-EtOAc (5:1) solvents. 5 μL of the extract was analyzed by LC-MS (LC-MS analysis conditions for α-ketoglutarate and pyruvate: Tosoh TSKgel Amide-80, 5 μM, 4.6 mm × 250 mm; mobile phase A: 0.1% formic acid aqueous solution; B: MeCN containg 0.1% formic acid; gradient conditions: 0–15 min, linear gradient 99–90% B; 16 min, 10% B; 26 min, 10% B; flow rate: 1 mL/min).

### General procedure for conversion of 3 to 1 and 2 in the fermentation medium, H_2_O and MeOH

Compound **3** (4 mg), α-ketoglutaric acid (46.7 mg), and pyruvic acid (28.2 mg) were separately dissolved in 0.5 mL of MeOH to make 6.4 mM, 640 mM, and 640 mM stock solutions, respectively. For conversion of **3** to **1**, stock solutions of **3** and α-ketoglutaric acid (50 μL each) were separately added to 200 μL of the M8 medium, H_2_O, and MeOH. Similarly, for conversion of **3** to **2**, stock solutions of **3** and pyruvic acid (50 μL each) were added to 200 μL of the M8 medium, H_2_O, and MeOH. After shaking at 200 rpm at 28 °C for 36 h, 0.4 mL of MeOH was added to the reaction mixture to get a clear solution. Aliquots (0.2 mL) were taken from each reaction and analyzed by LC-MS.

### Antibacterial Assay

The antibacterial assay was performed by using the agar dilution method as described previously^13^. Organisms used in this study included strains from the ATCC collection and clinical isolates. The test medium was Mueller-Hinton broth, and the inoculum was 10,000 colony forming units (CFU)/spot. The final concentrations of compounds ranged from 0.03 to 128 μg/mL. Culture plates were incubated at 35 °C for 18 h and MICs were then recorded. The MIC was defined as the lowest concentration that prevented visible growth of the bacteria.

### Data Availability

All data generated or analysed during this study are included in this published article (and its Supplementary Information files).

## Electronic supplementary material

Supplementary Information
