# Supplementary material for: Neo-actinomycins A and B, natural actinomycins bearing the 5H-oxazolo[4,5-b]phenoxazine chromophore, from the marine-derived Streptomyces sp. IMB094
Source: Sci Rep. 2017 Jun 15;7:3591. doi: 10.1038/s41598-017-03769-8 (PMC5472614; doi:10.1038/s41598-017-03769-8)
Supplement: Supplementary file 1 — Supplementary Information [file 41598_2017_3769_MOESM1_ESM.pdf]

Neo-actinomycins A and B, natural actinomycins bearing the 5*H*-oxazolo[4,5-*b*]phenoxazine chromophore, from the marine-derived *Streptomyces* sp. IMB094

Qiang Wang, Yixuan Zhang, Mian Wang, Yi Tan, Xinxin Hu, Hongwei He, Chunling Xiao, Xuefu You, Yiguang Wang, and Maoluo Gan*

Institute of Medicinal Biotechnology, Chinese Academy of Medical Sciences and Peking Union Medical College, Beijing 100050, China

E-mail: ganml@imb.pumc.edu.cn; ganml@hotmail.com

Supplementary Information

Contents

Table S1. ^{13}C NMR Spectroscopic Data for Neo-actinomycin (1) and Actinomycin D (3) in DMSO-d_6 ....S3
Table S2. ^{1}H NMR Spectroscopic Data for Neo-actinomycin (1) and Actinomycin D (3) in DMSO-d_6 ....S4
Table S3. ^{13}C NMR Spectroscopic Data for Neo-actinomycin B (2)..S5
Table S4. ^{1}H NMR Spectroscopic Data for Neo-actinomycin B (2)...S6
Figure S1. Typical HPLC chromatogram at 400 nm and UV spectra for crude extracts of strain IMB094 cultures under normal fermentation conditions. S7
Figure S2. LC-MS chromatograms of (a) L-FDLA and (b) L/D-FDLA derivatives of the hydrolyzate of **1** S8
Table S5. LC-MS Retention times ($t_{R,}$ minute) of the derivatizd amino acids from the hydrolysate of **1**.........................S8
Figure S3. Key HMBC and ^{1}H-^{1}H COSY correlations of **2**. S9
Figure S4. LC-MS analysis of neo-actinomycins in the cultures extracts of strain IMB094 in the feeding experiments .S9
Figure S5. LC-MS traces at 400 nm for (a) actinomycin D (**3**) and conversion of **3** to **1** with α-ketoglutaric acid in the fermentation M8 media (b), H_2O (c), and MeOH (d) S10
Figure S6. LC-MS traces at 400 nm for (a) actinomycin D (**3**) and conversion of **3** to **2** with pyruvic acid in the fermentation M8 media (b), H_2O (c), and MeOH (d) after 36 h. S10
Table S6. Conversion rates (%) of actinomycin D with α-ketoglutaric acid and pyruvic acid into neo-actinomycins A (**1**) and B (**2**) in MeOH and H_2O under different pH. S11
Figure S7. LC-MS analysis of actinomycin analogs in the cultures extracts of strain IMB094 grown in the M8 and GGM media……………………………………………………………………………………………………S11
Figure S8. LC-MS analysis of α-ketoglutarate and pyruvate in the cultures extracts of strain IMB094 and in the media prior to cultivation. ……………………………………………………………………………………………S12
Table S7. Concentrations of compounds **1–4**, α-ketoglutarate, and pyruvate in the fermentation broth cultured in M8 and GGM media…………………………………………………………………………………………………S12
Figure S9. The CD and UV spectra of compounds **1–3** recorded in MeOH. S13
Figure S10. The (+)-HRESIMS spectrum of neo-actinomycin A (**1**)..S14
Figure S11. The ^{1}H NMR spectrum of neo-actinomycin A (**1**) in DMSO-d_6 (600 MHz). S15
Figure S12. The ^{13}C NMR spectrum of neo-actinomycin A (**1**) in DMSO-d_6 (150 MHz). S16
Figure S13. The DEPT spectrum of neo-actinomycin A (**1**) in DMSO-d_6 (150 MHz). S17
Figure S14. The COSY spectrum of neo-actinomycin A (**1**) in DMSO-d_6 (600 MHz)..S18
Figure S15. The HSQC spectrum of neo-actinomycin A (**1**) in DMSO-d_6 (600 MHz)..S19
Figure S16. The HMBC spectrum of neo-actinomycin A (**1**) in DMSO-d_6 (600 MHz)...S20
Figure S17. The ROESYspectrum of neo-actinomycin A (**1**) in DMSO-d_6 (600 MHz). S21
Figure S18. The (+)-HRESIMS spectrum of neo-actinomycin B (**2**)...S22
Figure S19. The ^{1}H NMR spectrum of neo-actinomycin B (**2**) in DMSO-d_6 (600 MHz). S23
Figure S20. The ^{13}C NMR spectrum of neo-actinomycin B (**2**) in DMSO-d_6 (150 MHz). S24
Figure S21. The ^{1}H NMR spectrum of neo-actinomycin B (**2**) in MeOH-d_4 (600 MHz)...S25
Figure S22. The ^{13}C NMR spectrum of neo-actinomycin B (**2**) in MeOH-d_4 (150 MHz)..S26
Figure S23. The DEPT spectrum of neo-actinomycin B (**2**) in MeOH-d_4 (150 MHz). S27
Figure S24. The COSY spectrum of neo-actinomycin B (**2**) in MeOH-d_4 (600 MHz). S28
Figure S25. The TOCSY spectrum of neo-actinomycin B (**2**) in MeOH-d_4 (600 MHz)..S29
Figure S26. The HSQC spectrum of neo-actinomycin B (**2**) in MeOH-d_4 (600 MHz). S30
Figure S27. The HMBC spectrum of neo-actinomycin B (**2**) in MeOH-d_4 (600 MHz). S31
Figure S28. The ROESY spectrum of neo-actinomycin B (**2**) in MeOH-d_4 (600 MHz). S32

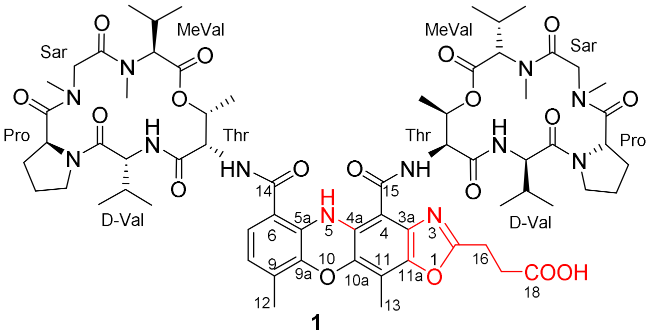

1

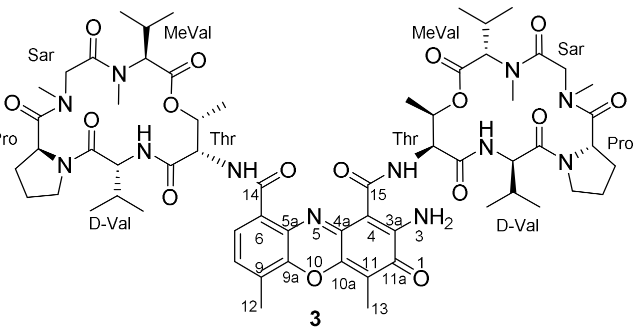

3

Table S1. ^{13}**C NMR Spectroscopic Data for Neo-actinomycin (1) and Actinomycin D (3) in DMSO-d_6**[a]

no.	pentapeptidolactone (α-ring)			no.	pentapeptidolactone (β-ring)		
	1	3	Δδ		1	3	Δδ
Thr 1	169.2	168.2	1.0	Thr 1	169.4	168.3	1.1
2	53.9	54.4	-0.5	2	54.9	54.4	0.5
3	72.3	73.5	-1.2	3	71.9	73.5	-1.6
4	16.3	16.9	-0.6	4	16.7	16.9	-0.2
Val 1	170.6	172.7	-2.1	Val 1	170.7	172.7	-2.0
2	57.4	57.6	-0.2	2	57.4	57.7	-0.3
3	30.1	30.8	-0.7	3	30.2	30.8	-0.6
4	18.6	18.6	0.0	4	18.6	18.6	0.0
5	19.1	19.1	0.0	5	19.1	19.1	0.0
Pro 1	173.0	172.3	0.7	Pro 1	173.0	172.3	0.7
2	54.8	55.9	-1.1	2	54.8	55.9	-1.1
3	31.1	30.5	0.6	3	31.2	30.5	0.7
4	22.7	22.5	0.2	4	22.8	22.5	0.3
5	46.4	46.8	-0.4	5	46.4	46.8	-0.4
Sar 1	167.0	166.7	0.3	Sar 1	167.1	166.9	0.2
2	51.2	50.9	0.3	2	51.2	50.9	0.3
NMe	34.4	34.3	0.1	NMe	34.4	34.3	0.1
MeVal 1	168.1	168.1	0.0	MeVal 1	168.2	168.1	0.1
2	69.5	69.3	0.2	2	69.6	69.4	0.2
3	26.5	26.7	-0.2	3	26.5	26.7	-0.2
4	21.0	21.1	-0.1	4	21.1	21.1	0.0
5	18.8	18.6	0.2	5	18.8	18.6	0.2
NMe	38.6	38.5	0.1	NMe	38.8	38.5	0.3
chromophore							
2	165.8			10a	139.5	144.7	-5.2
3a	132.9	146.5	-13.6	11	111.8	111.9	-0.1
4	100.2	102.2	-2.0	11a	143.5	178.7	-35.2
4a	132.0	145.5	-13.5	12	14.8	14.6	0.2
5a	131.3	129.3	2.0	13	8.9	7.6	1.3
6	113.4	131.6	-18.2	14	166.6	166.1	0.5
7	122.0	125.1	-3.1	15	165.4	165.9	-0.5
8	122.0	129.6	-7.6	16	23.3		
9	127.5	127.0	0.5	17	30.3		
9a	140.7	140.0	0.7	18	173.1		

[a] ^{13}C NMR data were recorded at 150 MHz. For comparison of NMR data of **1** and **3**, the atom numbers in **3** are labelled as the same as **1**.

Table S2. ^{1}**H NMR Spectroscopic Data for Neo-actinomycin (1) and Actinomycin D (3) in DMSO-d_6**[a]

no.	pentapeptidolactone (α-ring)		no.	pentapeptidolactone (β-ring)	
	1	3		1	3
Thr 2	4.97, dd (9.0, 2.4)	4.50, dd (7.2, 2.4)	Thr 2	4.84, dd (8.4, 2.4)	4.50, dd (7.2, 2.4)
3	5.14, qd (6.0, 2.4)	5.07, m	3	5.24, qd (6.0, 2.4)	5.07, m
4	1.17, d (6.0)	1.18, d (6.6)	4	1.20, d (6.0)	1.18, d (6.6)
NH	7.25, d (9.0)		NH	9.66, d (8.4)	
Val 2	3.50, m	3.55, m	Val 2	3.49, m	3.55, m
3	1.88, m	1.96, m	3	1.88, m	1.96, m
4	0.92, d (6.6)	0.91, d (6.0)	4	0.94, d (6.6)	0.91, d (6.0)
5	0.69, d (7.2)	0.71, d (6.6)	5	0.70, (7.2)	0.71, d (6.6)
NH	8.42, d (6.0)		NH	8.29, d (5.4)	
Pro 2	6.24, dd (9.0, 3.0)	6.06, brd (10.2)	Pro 2	6.21, dd (9.0, 3.0)	6.05, brd (10.2)
3	2.10, m; 1.74, m	2.43, m; 1.73, m	3	2.10, m,1.74, m	2.43, m; 1.73, m
4	1.91, m; 1.67, m	2.00, m; 1.96, m	4	1.91, m,1.67, m	2.00, m; 1.96, m
5	3.49, m; 3.27, m	3.81, m; 3.50, m	5	3.50, m,3.27, m	3.81, m; 3.50, m
Sar 2	4.80, d (18.0); 4.08, d (18.0)	4.56, d (18.0); 4.04, d (18.0)	Sar 2	4.78, d (18.0); 4.08, d (18.0)	4.56, d (18.0); 4.04, d (18.0)
NMe	2.75, s	2.72, s	NMe	2.75, s	2.72, s
MeVal 2	3.23, d (9.6)	3.13, d (9.6)	MeVal 2	3.23, d (9.6)	3.07, d (9.6)
3	2.54, m	2.46, m	3	2.54, m	2.46, m
4	0.97, d (6.6)	0.97, d (6.6)	4	0.98, d (6.6)	0.97, d (6.6)
5	0.79, d (6.6)	0.74, d (6.6)	5	0.80, d (6.6)	0.74, d (6.6)
NMe	3.19, s	2.94, s	NMe	3.19, s	2.92, s
chromophore					
5	11.74, s		13	2.27, s	2.50, s
7	7.11, d (8.4)	7.41, d (7.8)	16	3.17, m	
8	6.62, d (8.4)	7.41, d (7.8)	17	2.89, m	
12	2.13, s	2.14, s			

[a] ^{1}H NMR data were recorded at 600 MHz. For comparison of NMR data of **1** and **3**, the atom numbers in **3** are labelled as the same as **1**.

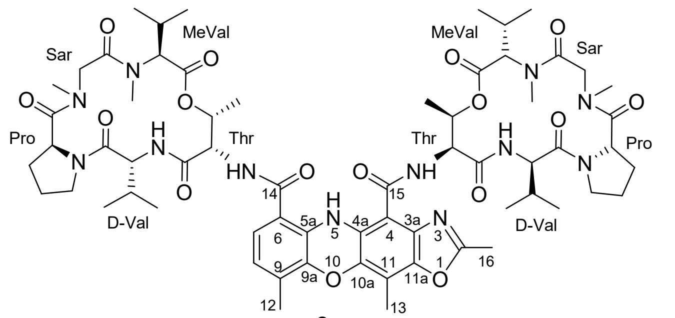

2

Table S3. ^{13}C NMR Spectroscopic Data for Neo-actinomycin B (2)[a]

no.	pentapeptidolactone (α-ring)		no.	pentapeptidolactone (β-ring)	
	DMSO-d_6	MeOH-d_4		DMSO-d_6	MeOH-d_4
Thr 1	169.2, C	171.3, C	Thr 1	169.4, C	171.6, C
2	54.0, CH	55.8, CH	2	54.8, CH	56.2, CH
3	72.2, CH	74.6, CH	3	71.9, CH	74.0, CH
4	16.4, CH_3	17.3, CH_3	4	16.8, CH_3	17.2, CH_3
Val 1	170.6, C	173.8, C	Val 1	170.8, C	173.9, C
2	57.4, CH	59.6, CH	2	57.4, CH	59.5, CH
3	30.1, CH	32.0, CH	3	30.2, CH	32.0, CH
4	18.7, CH_3	19.8, CH_3	4	18.6, CH_3	19.8, CH_3
5	19.1, CH_3	19.5, CH_3	5	19.1, CH_3	19.5, CH_3
Pro 1	173.0, C	175.5, C	Pro 1	173.1, C	175.7, C
2	54.7, CH	57.6, CH	2	54.4, CH	57.4, CH
3	31.3, CH_2	32.5, CH_2	3	31.2, CH_2	32.5, CH_2
4	22.8, CH_2	24.1, CH_2	4	22.8, CH_2	24.1, CH_2
5	46.4, CH_2	48.4, CH_2	5	46.4, CH_2	48.3, CH_2
Sar 1	167.2, C	168.7, C	Sar 1	167.0, C	168.6, C
2	51.3, CH_2	53.0, CH_2	2	51.1, CH_2	52.9, CH_2
NMe	34.4, CH_3	35.7, CH_3	3	34.4, CH_3	35.7, CH_3
MeVal 1	168.2, C	169.7, C	MeVal 1	168.3, C	169.8, C
2	69.5, CH	72.1, CH	2	69.5, CH	72.4, CH
3	26.5, CH	28.2, CH	3	26.3, CH	27.9, CH
4	21.0, CH_3	21.7, CH_3	4	21.1, CH_3	21.7, CH_3
5	18.8, CH_3	19.4, CH_3	5	18.8, CH_3	19.4, CH_3
NMe	38.6, CH_3	39.6, CH_3	NMe	38.6, CH_3	40.1, CH_3
chromophore					
2	165.5, C	164.5, C	9a	140.7, C	142.8, C
3a	133.2, C	135.1, C	10a	139.4, C	141.5, C
4	100.1, C	101.7, C	11	111.7, C	113.5, C
4a	132.1, C	134.1, C	11a	143.7, C	145.6, C
5a	131.4, C	132.8, C	12	14.8, CH_3	15.4, CH_3
6	113.5, C	115.6, C	13	8.9, CH_3	9.2, CH_3
7	122.0, CH	122.3, CH	14	166.6, C	168.8, C
8	122.1, CH	123.4, CH	15	163.7, C	167.7, C
9	127.5, C	129.1, C	16	14.4, CH_3	14.5, CH_3

[a] ^{13}C NMR data were recorded at 600 MHz.

Table S4. ^{1}**H NMR Spectroscopic Data for Neo-actinomycin B (2)**[a]

	pentapeptidolactone (α-ring)			pentapeptidolactone (β-ring)	
no.	DMSO-d_6	MeOH-d_4	no.	DMSO-d_6	MeOH-d_4
Thr 2	4.94, brd (9.6)	4.95, d (2.4)	Thr 2	4.94, brd (9.6)	5.01, d (2.4)
3	5.15, qd (6.6, 2.4)	5.33, d (6.0, 2.4)	3	5.23, qd (6.6, 2.4)	5.39, qd (6.0, 2.4)
4	1.17, d (6.6)	1.31, d (6.0)	4	1.18, d (6.6)	1.29, d (6.0)
NH	7.28, d (9.6)		NH	9.64, d (9.6)	
Val 2	3.51, m	3.70, d (10.2)	Val 2	3.51, m	3.66, d (10.2)
3	1.86, m	2.04, m	3	1.86, m	2.06, m
4	0.93, d (6.6)	1.08, d (6.6)	4	0.94, d (6.6)	1.08, d (6.6)
5	0.69, d (6.6)	0.86, d (6.0)	5	0.70, d (6.6)	0.85, d (6.6)
NH	8.36, d (6.0)		NH	8.31, d (6.0)	
Pro 2	6.25, dd (9.0, 3.6)	6.42, dd (9.0, 3.6)	Pro 2	6.21, dd (9.0, 3.6)	6.48, dd (9.0, 3.6)
3	2.09, m; 1.74, m	2.28, m; 1.94, m	3	2.09, m; 1.74, m	2.24, m; 1.90, m
4	1.90, m; 1.67, m	2.05, m; 1.83, m	4	1.90, m; 1.67, m	2.05, m; 1.83, m
5	3.48, m	3.61, m; 3.51, m	5	3.48, m	3.59, m; 3.49, m
Sar 2	4.82, d (18.0)	4.96, d (17.4)		4.78, d (18.0)	4.90, d (18.0)
	4.08, d (18.0)	4.12, d (17.4)	Sar 2	4.06, d (18.0)	4.09, d (18.0)
NMe	2.75, s	2.92, s	3	2.75, s	2.91, s
MeVal 2	3.23, d (9.6)	3.19, d (9.0)	MeVal 2	3.25, d (9.6)	3.21, d (9.0)
3	2.58, m	2.73, m	3	2.55, m	2.67, m
4	1.00, d (6.6)	1.04, d (6.0)	4	0.97, d (6.6)	1.06, d (6.0)
5	0.79, d (6.6)	0.88, d (6.0)	5	0.80, d (6.6)	0.89, d (6.0)
NMe	3.19, s	3.26, s	NMe	3.27, s	3.37, s
chromophore					
5	11.79, s		12	2.15, s	2.10, s
7	7.13, d (8.4)	6.96, d (8.4)	13	2.28, s	2.26, s
8	6.63, d (8.4)	6.51, d (8.4)	16	2.63, s	2.58, s

[a] ^{1}H NMR data were recorded at 600 MHz.

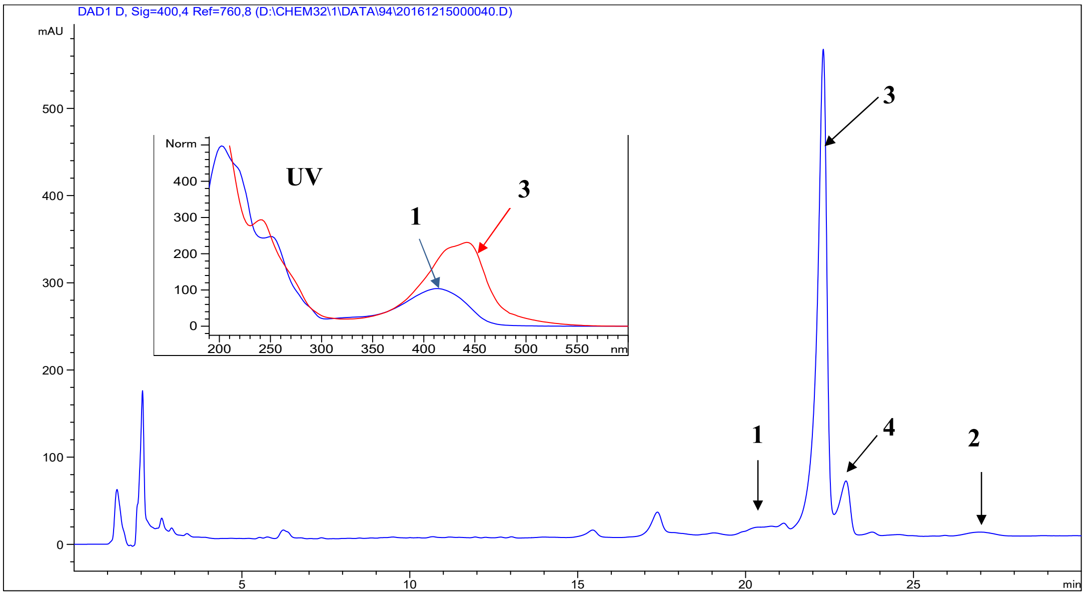

Figure S1. Typical HPLC chromatogram at 400 nm and UV spectra for crude extracts of strain IMB094 cultures under normal fermentation conditions.

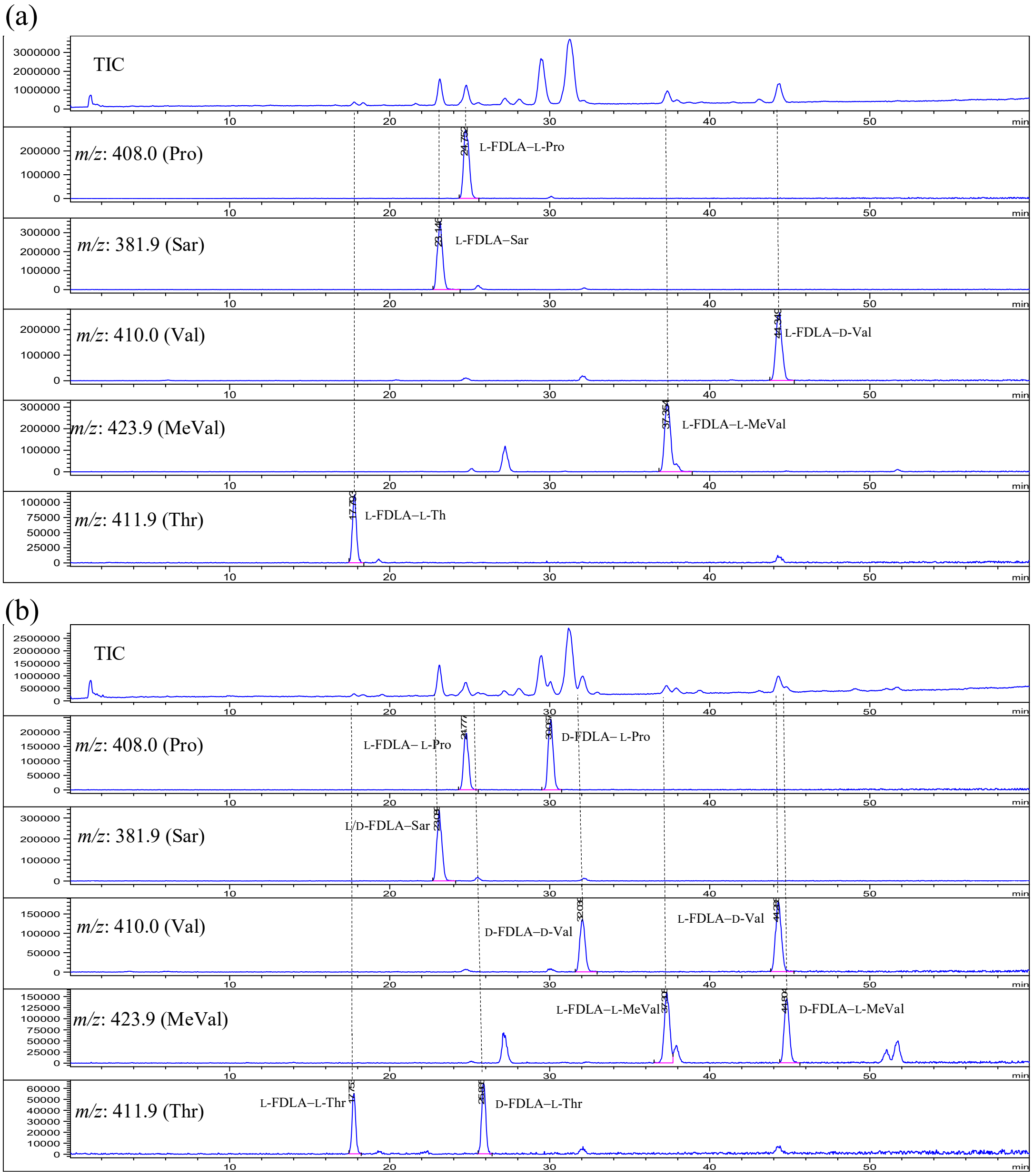

Figure S2. LC-MS chromatograms of (a) L-FDLA and (b) L/D-FDLA derivatives of the hydrolyzate of **1** recorded at negative ion mode

Table S2. LC-MS RetentionTimes (t_{R}, minute) of the Derivatizd Amino Acids from the Hydrolysate of 1.

	m/z [M-H]$^-$	L-FDLA-derivate	L/D-FDLA-derivate
L-Pro	408.0	24.75	24.78
			30.07
Sar	381.9	23.15	23.09
D-Val	410.0	44.35	32.04
			44.29
L-MeVal	423.9	37.35	37.31
			44.80
L-Thr	411.9	17.79	17.75
			25.87

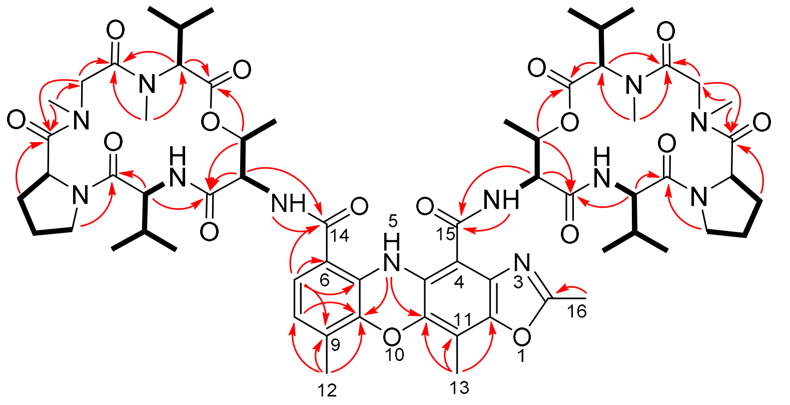

2

Figure S3. Key HMBC and ^{1}H-^{1}H COSY correlations of **2**.

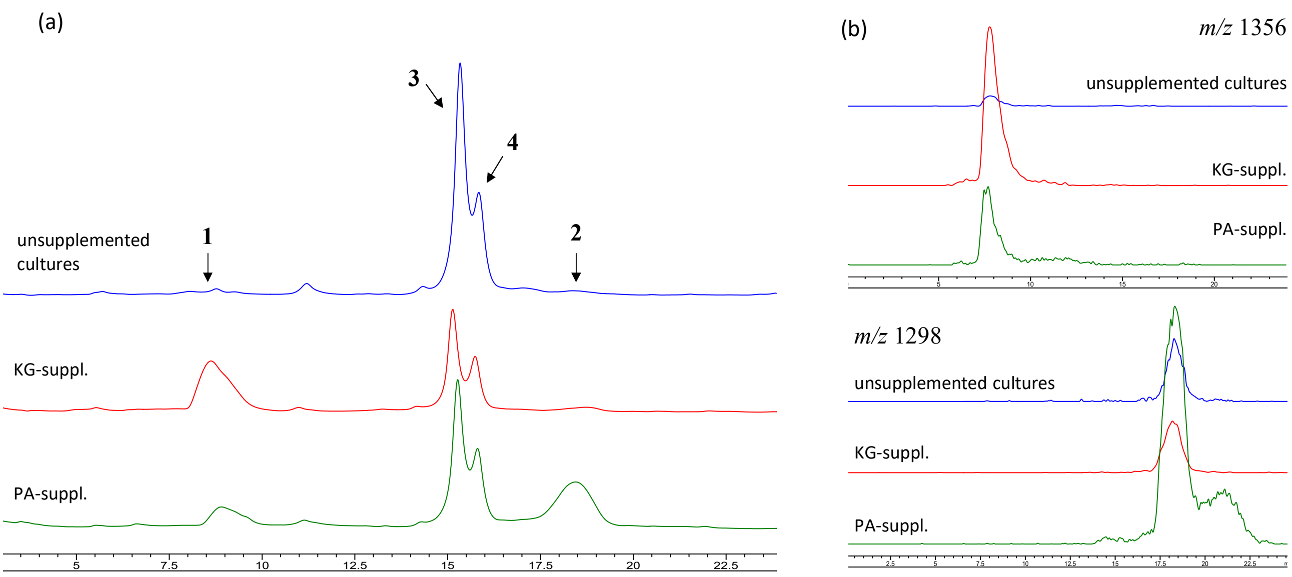

Figure S4. LC-MS analysis of neo-actinomycins in the cultures extracts of strain IMB094 in the feeding experiments (a) Chromatograms at 400 nm for unsupplemented cultures; cultures supplemented with 1mg/mL of α-ketoglutaric acid (KG) and cultures supplemented with 1mg/mL of pyruvic acid (PA); (b) Extracted ion chromatographs are shown for neo-actinomycins A (**1**, $[M+NH_4]^+$ = 1356) and B (**2**, $[M+NH_4]^+$ = 1298)

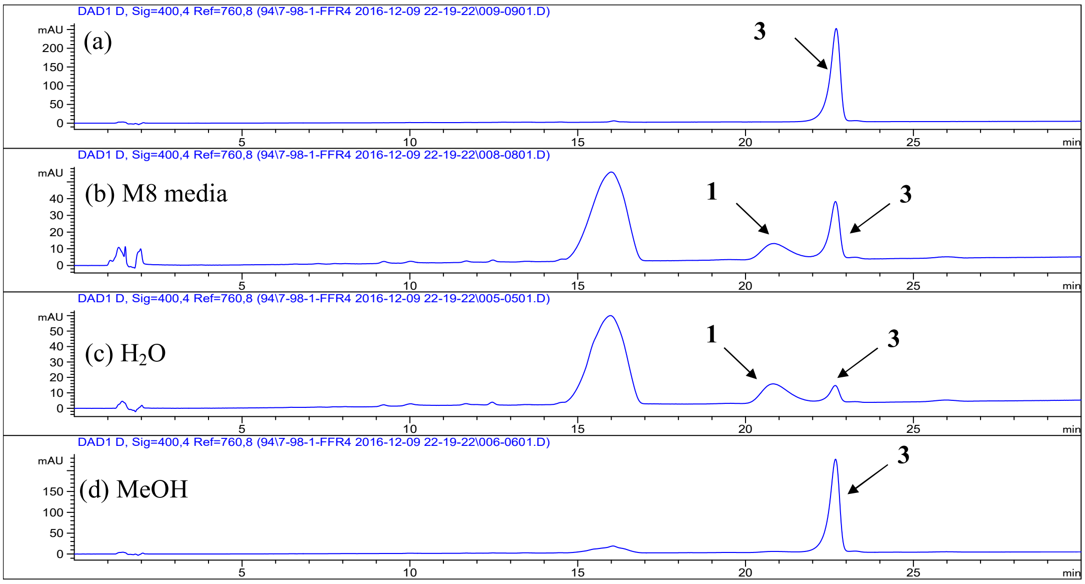

Figure S5. LC-MS traces at 400 nm for (a) actinomycin D (**3**) and conversion of **3** to **1** with α-ketoglutaric acid in the fermentation M8 media (b), H_2O (c), and MeOH (d) (LC-MS condition: Capcell MGII C-18 3μM, 3.0 mm × 150 mm; mobile phase A: H_2O containing 0.1% formic acid; B: MeCN containing 0.1% formic acid; gradient conditions: 0–30 min linear gradient 35–80% B; flow rate: 0.5 mL/min)

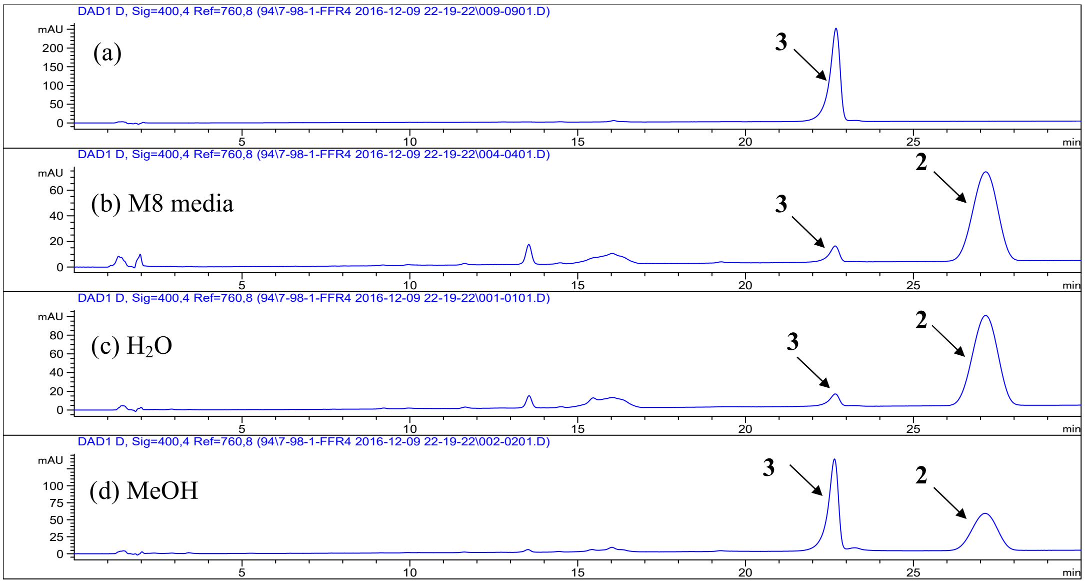

Figure S6. LC-MS traces at 400 nm for (a) actinomycin D (**3**) and conversion of **3** to **2** with pyruvic acid in the fermentation M8 media (b), H_2O (c), and MeOH (d) after 36 h. (LC-MS condition: Capcell MGII C-18 3μM, 3.0 mm × 150 mm; mobile phase A: H_2O containing 0.1% formic acid; B: MeCN containing 0.1% formic acid; gradient conditions: 0–30 min linear gradient 35–80% B; flow rate: 0.5 mL/min)

Table S6. Conversion rates (%) of actinomycin D with α-ketoglutaric acid and pyruvic acid into neo-actinomycins A (**1**) and B (**2**) in MeOH and H_2O under different pH [a]

pH condition	**1**		**2**	
	MeOH	H_2O	MeOH	H_2O
pH 1.0	0%	14.9%	4.0%	36.6%
pH 2.0	0%	18.7%	5.1%	52.3%
pH 4.0	0%	0%	0.8%	3.1%

[a] The pH 2.0 is the original pH value of the reaction mixtures (actinomycin D with α-ketoglutaric acid or pyruvic acid) wihout adjusting. The pH 1.0 and 4.0 was adjusted using 5 M HCl and 5 M ammonium hydroxide, respectively.

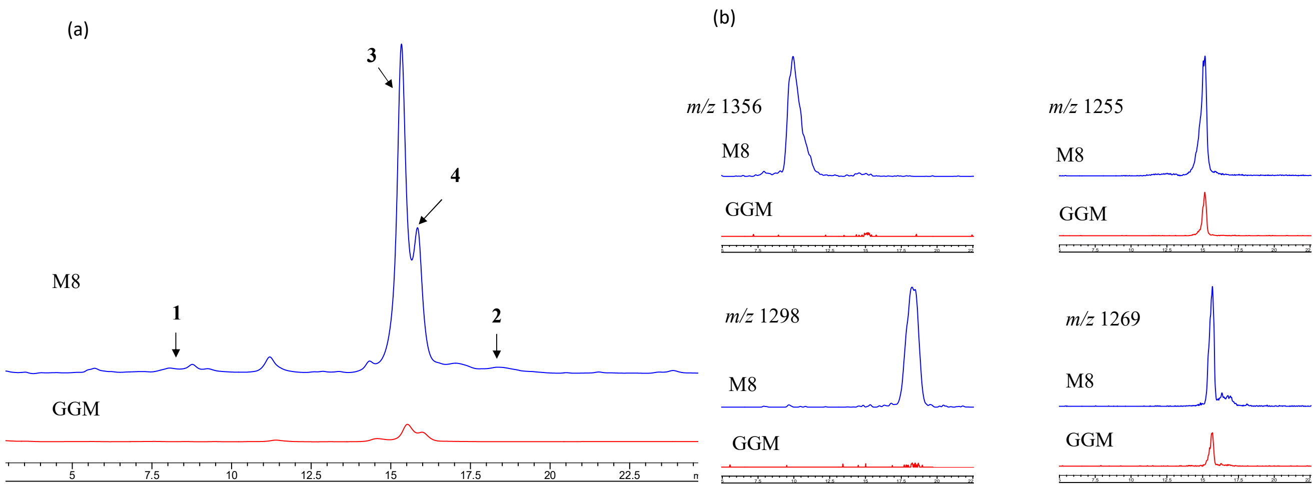

Figure S7. LC-MS analysis of actinomycin analogs in the cultures extracts of strain IMB094 grown in the M8 and GGM media. (a) Chromatograms at 400 nm; (b) Extracted ion chromatographs are shown for neo-actinomycins A (**1**, $[M+NH_4]^+$ = 1356), B (**2**, $[M+NH_4]^+$ = 1298), actinomycins D (**3**, $[M+H]^+$ = 1255) and X2 (**4**, $[M+H]^+$ = 1269).

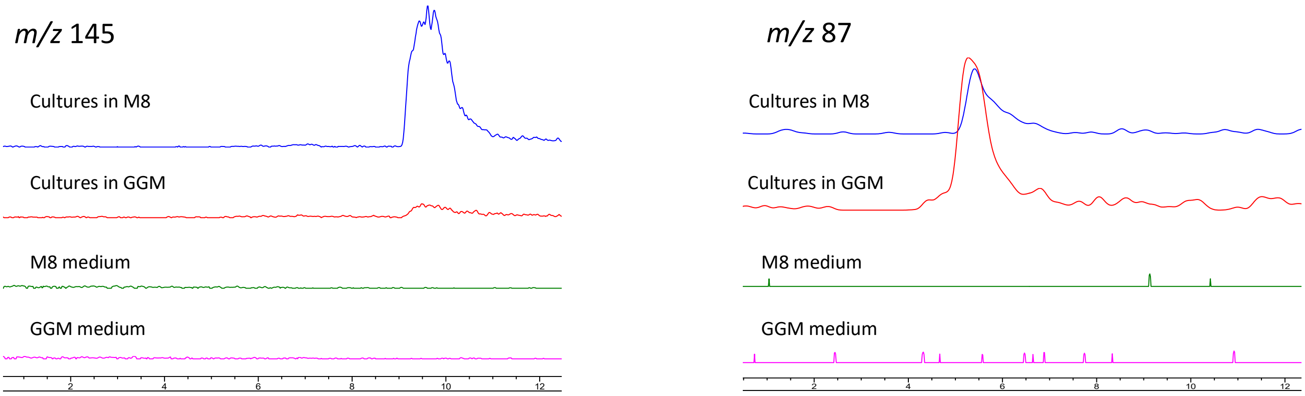

Figure S8. LC-MS analysis of α-ketoglutarate and pyruvate in the cultures extracts of strain IMB094 and in the media prior to cultivation. Extracted ion chromatographs are shown for α-ketoglutarate ($[M–H]^-$ = 145) and pyruvate ($[M–H]^-$ = 87).

Table S7. Concentrations of compounds **1**–**4**, α-ketoglutarate, and pyruvate in the fermentation broth cultured in M8 and GGM media (μg/mL)

Media	**1**	**2**	**3**	**4**	α-ketoglutarate	pyruvate
M8	12.2	3.7	39.9	23.3	39.4	7.7
GGM	0.0	0	2.8	1.6	3.9	21.4

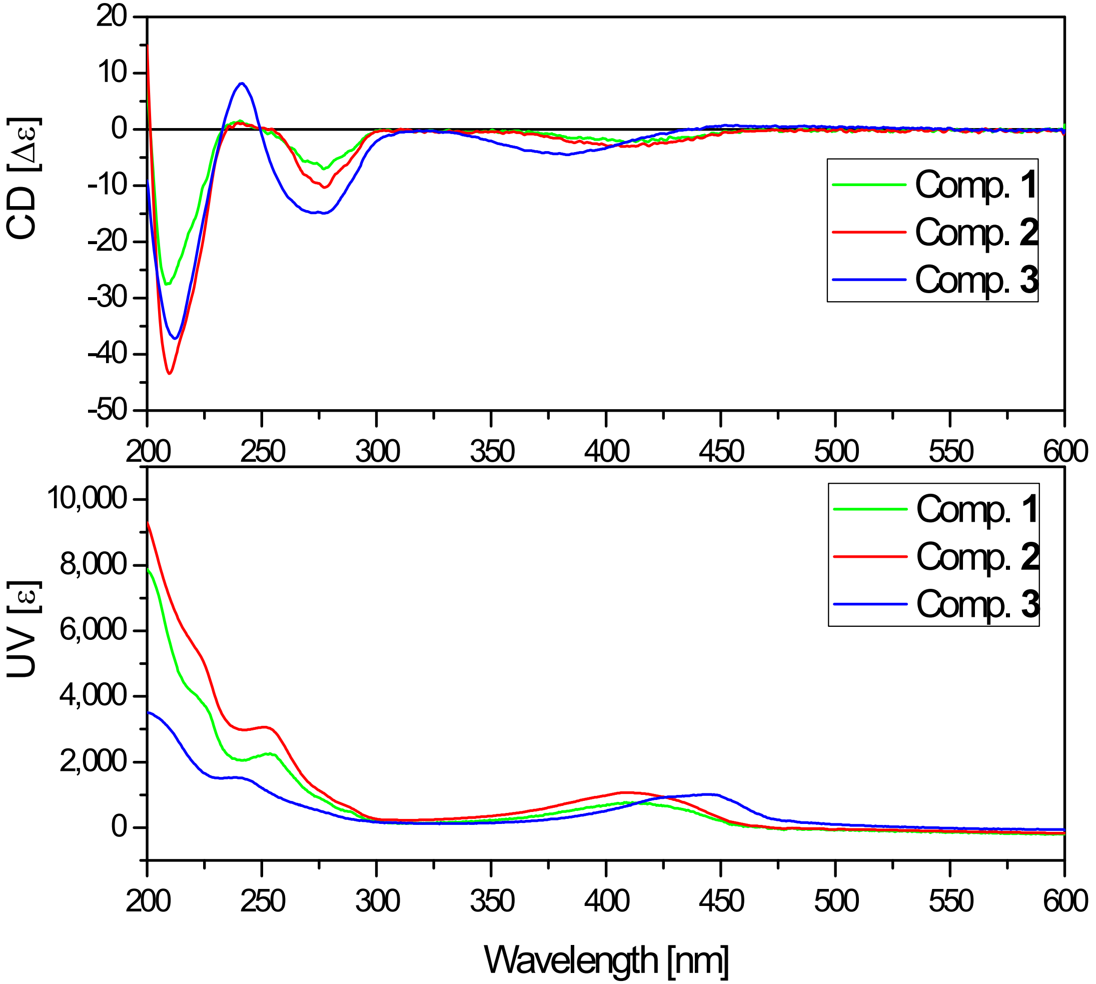

Figure S9. The CD and UV spectra of compounds **1**–**3** recorded in MeOH.

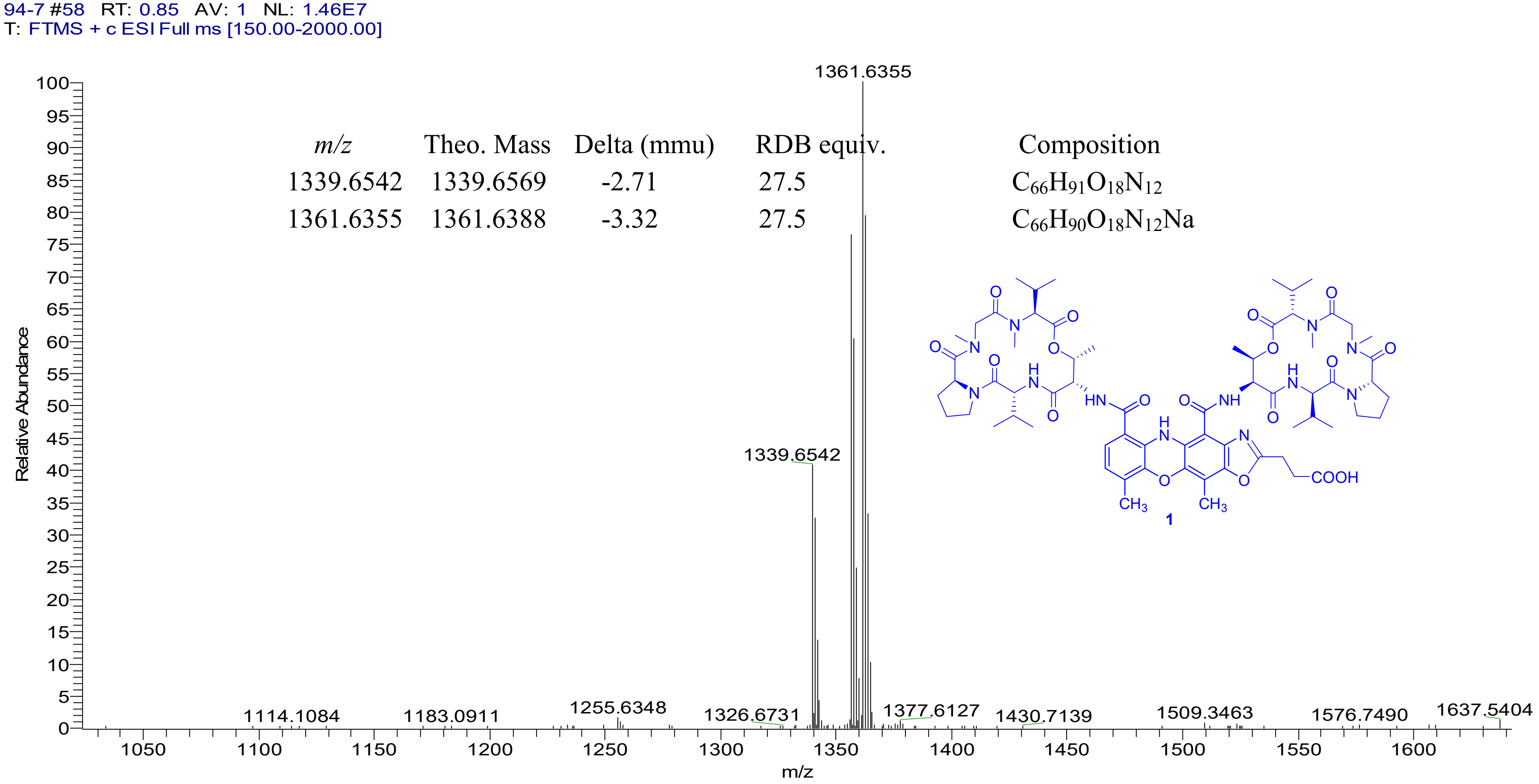

m/z	Theo. Mass	Delta (mmu)	RDB equiv.	Composition
1339.6542	1339.6569	-2.71	27.5	$C_{66}H_{91}O_{18}N_{12}$
1361.6355	1361.6388	-3.32	27.5	$C_{66}H_{90}O_{18}N_{12}Na$

Figure S10. The (+)-HRESIMS spectrum of neo-actinomycin A (**1**).

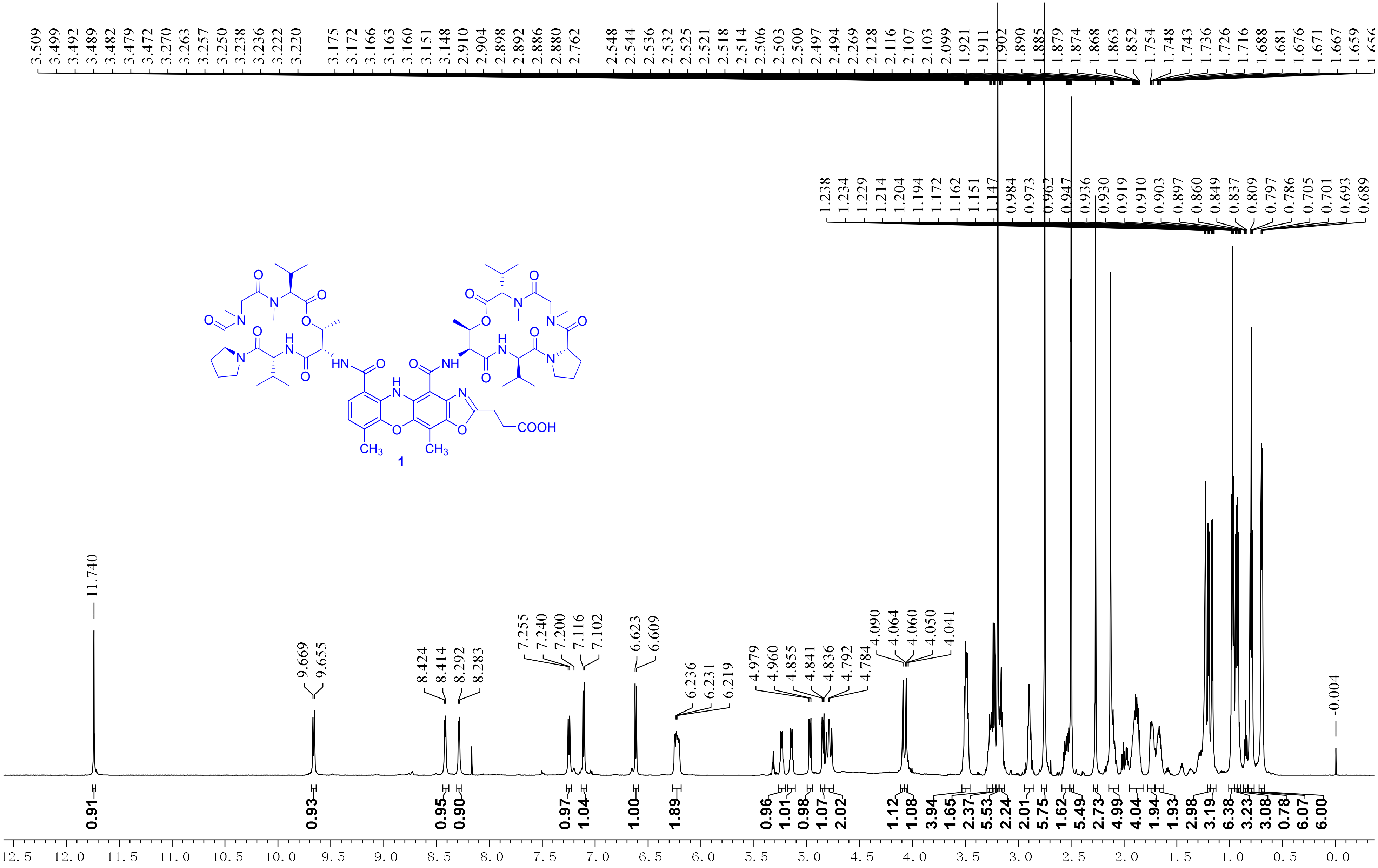

Figure S11. The ^{1}H NMR spectrum of neo-actinomycin A (**1**) in DMSO-d_6 (600 MHz).

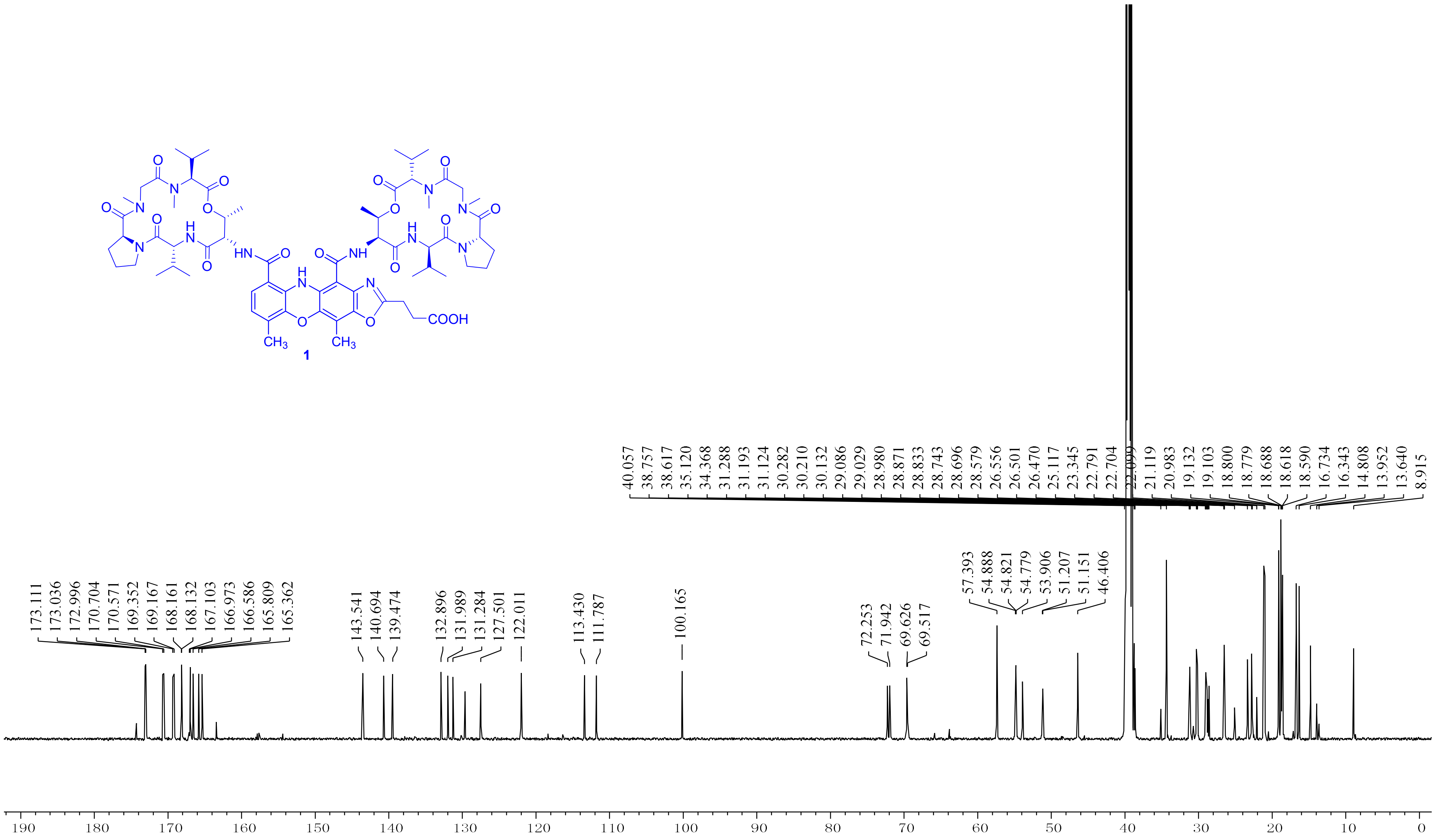

Figure S12. The ^{13}C NMR spectrum of neo-actinomycin A (**1**) in DMSO-d_6 (150 MHz).

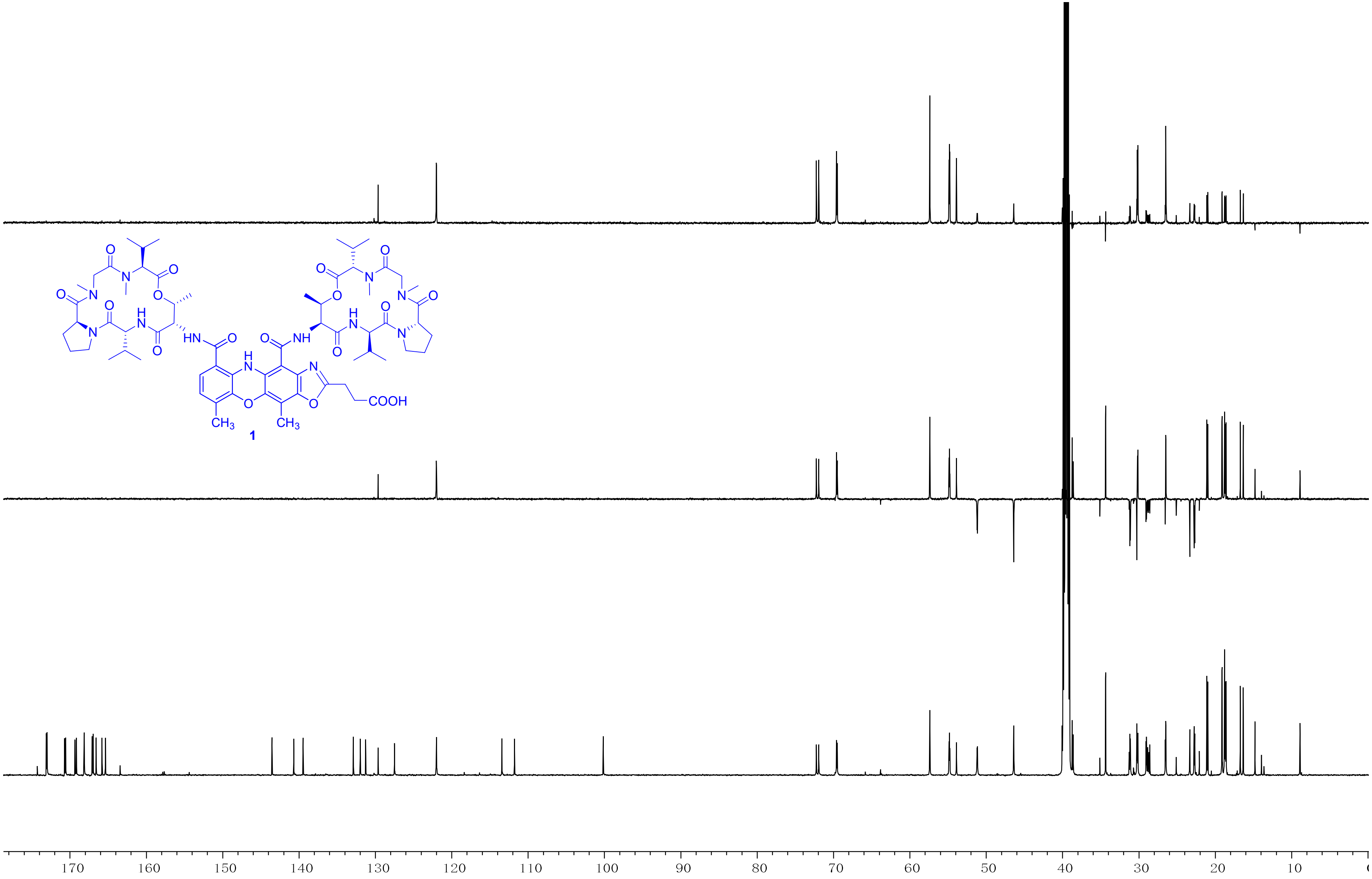

Figure S13. The DEPT spectrum of neo-actinomycin A (**1**) in DMSO-d_6 (150 MHz).

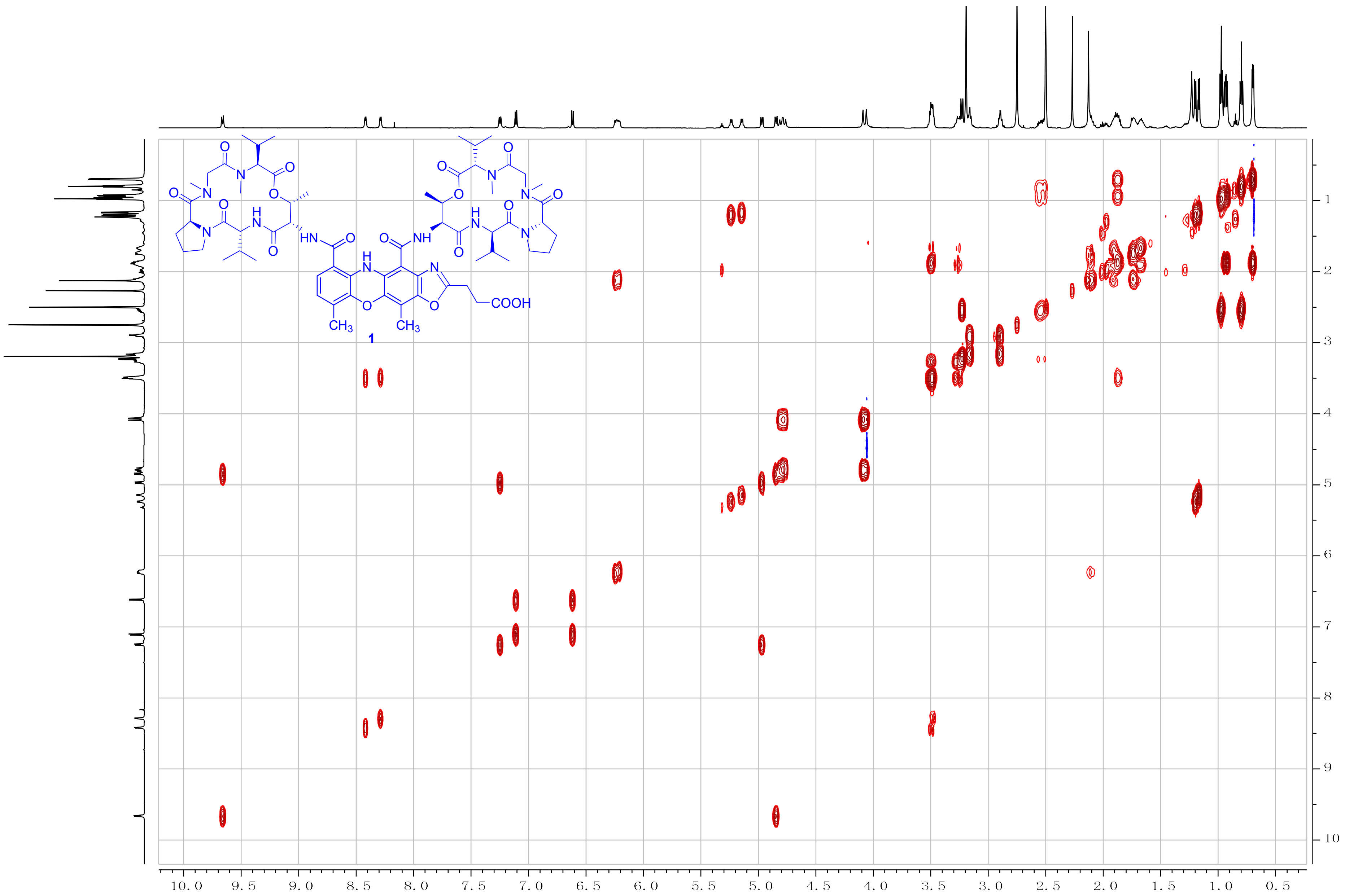

Figure S14. The COSY spectrum of neo-actinomycin A (**1**) in DMSO-d_6 (600 MHz).

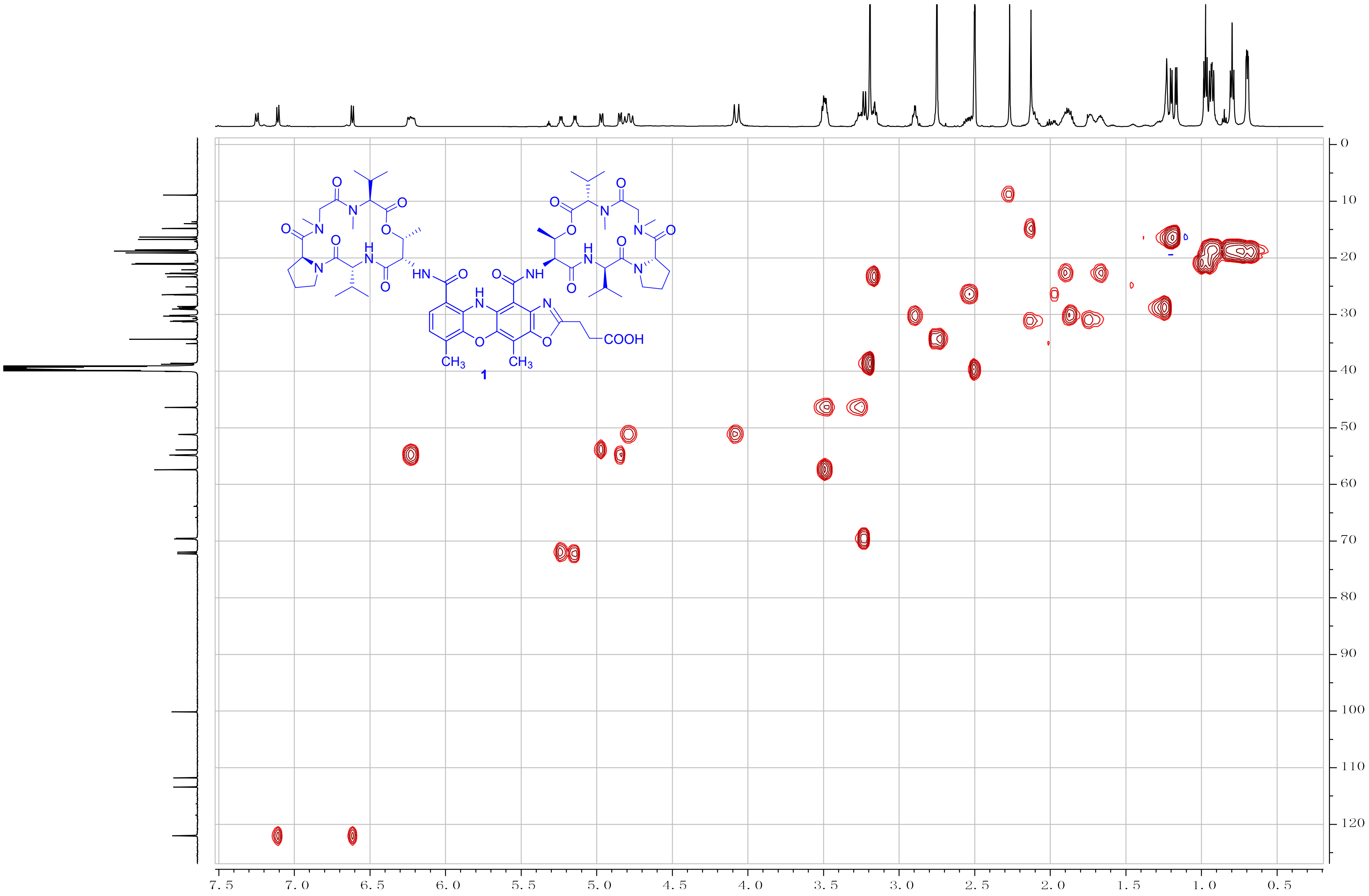

Figure S15. The HSQC spectrum of neo-actinomycin A (**1**) in DMSO-d_6 (600 MHz).

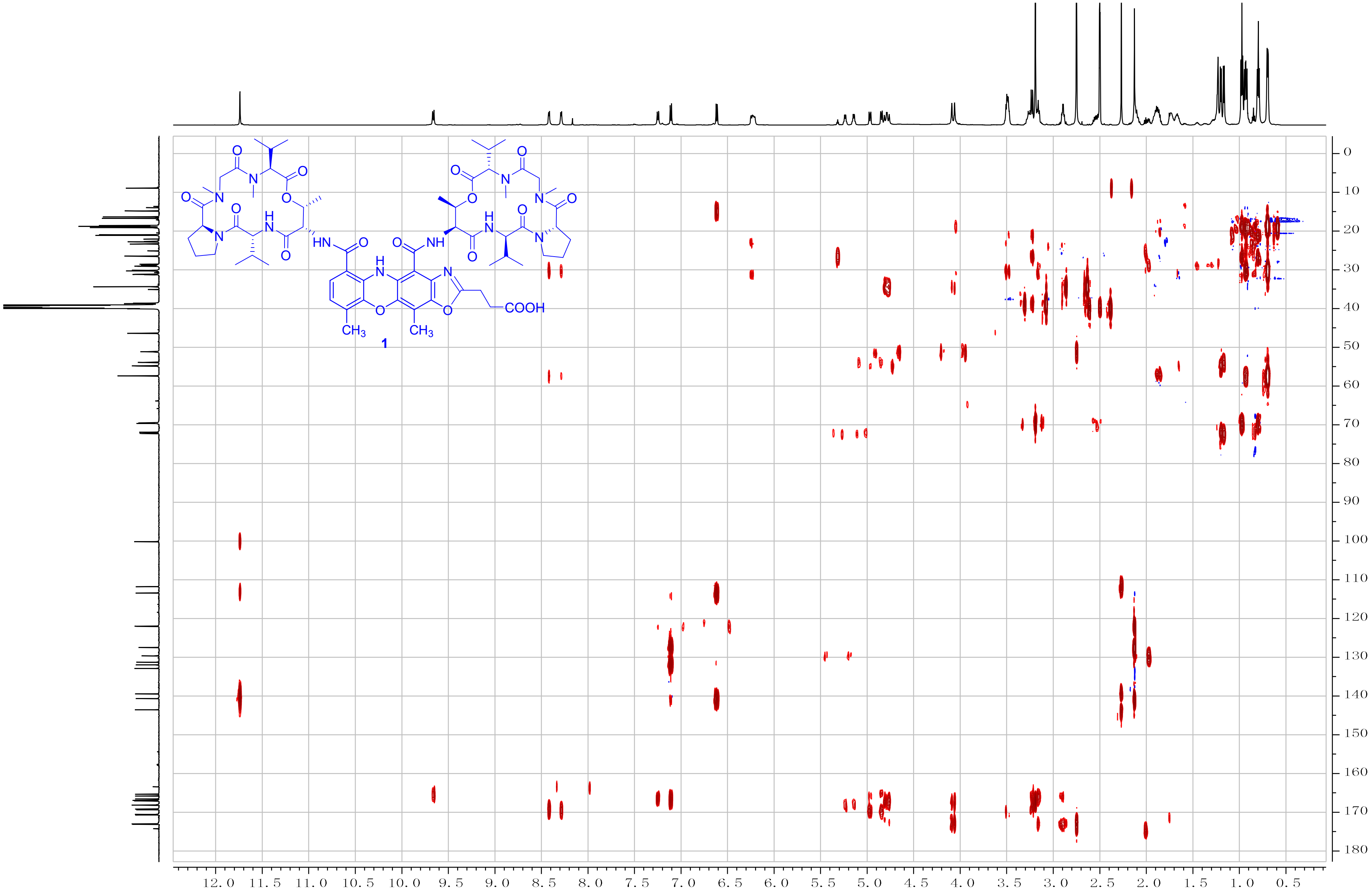

Figure S16. The HMBC spectrum of neo-actinomycin A (**1**) in DMSO-d_6 (600 MHz).

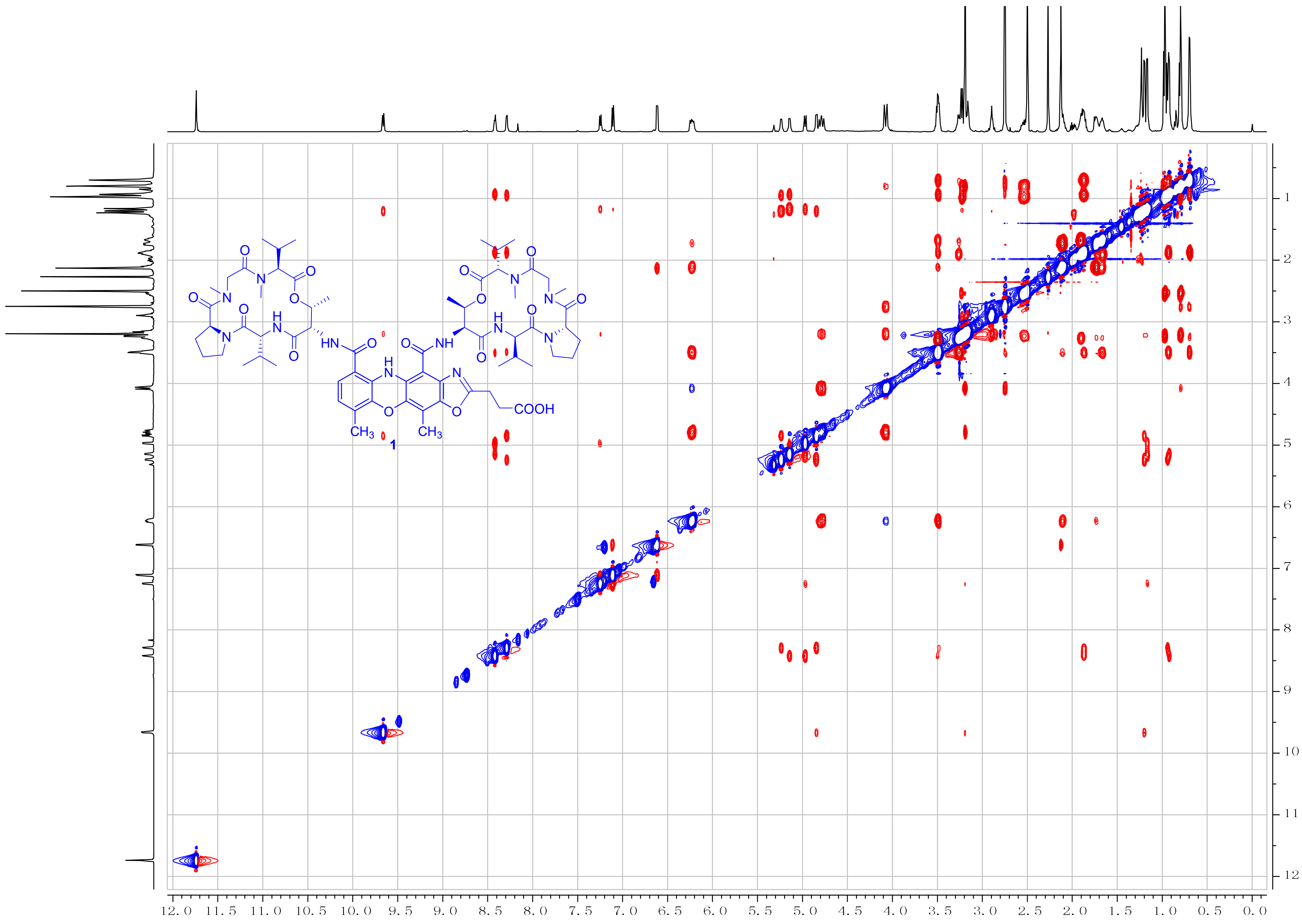

Figure S17. The ROESYspectrum of neo-actinomycin A (**1**) in DMSO-d_6 (600 MHz).

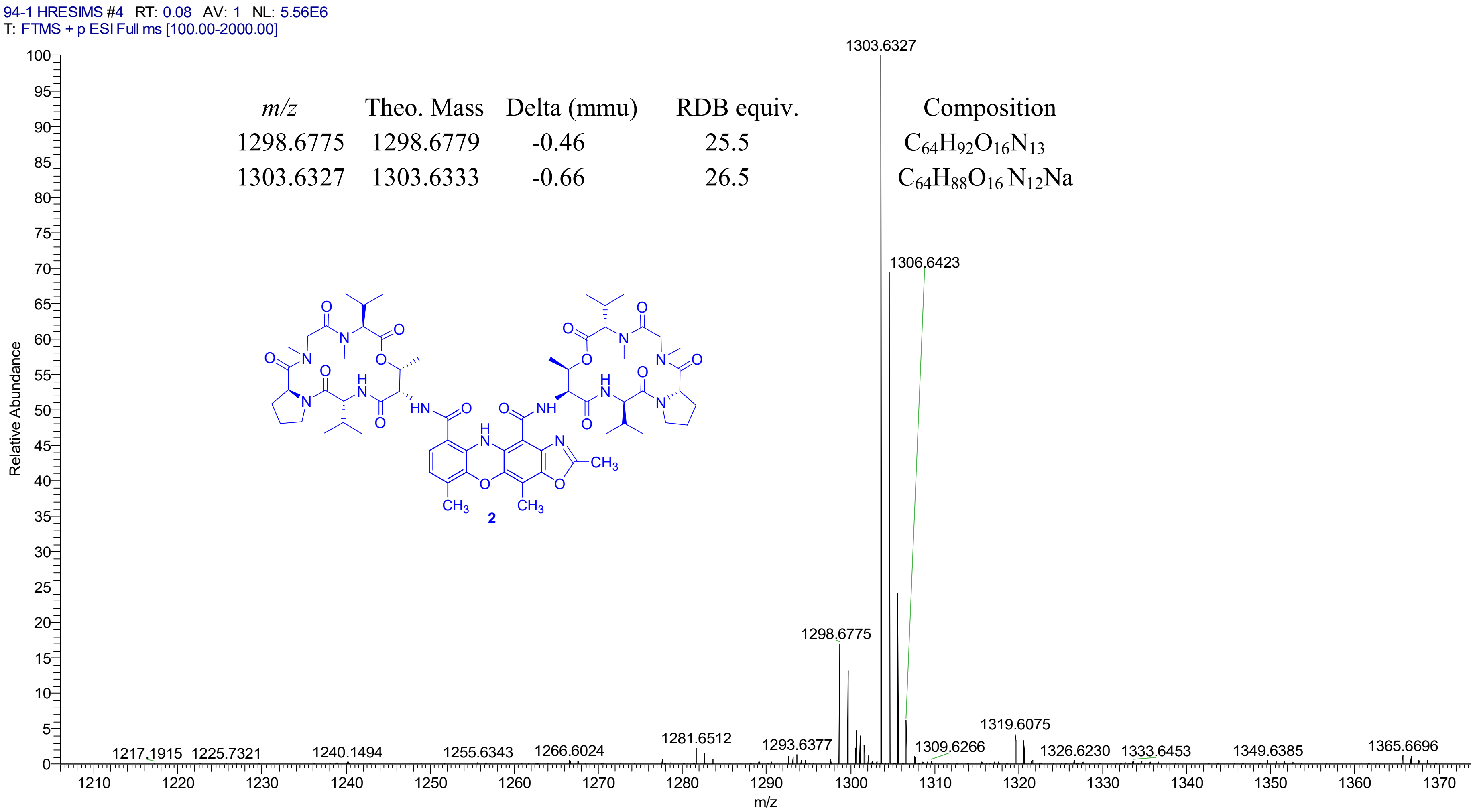

Figure S18. The (+)-HRESIMS spectrum of neo-actinomycin B (**2**).

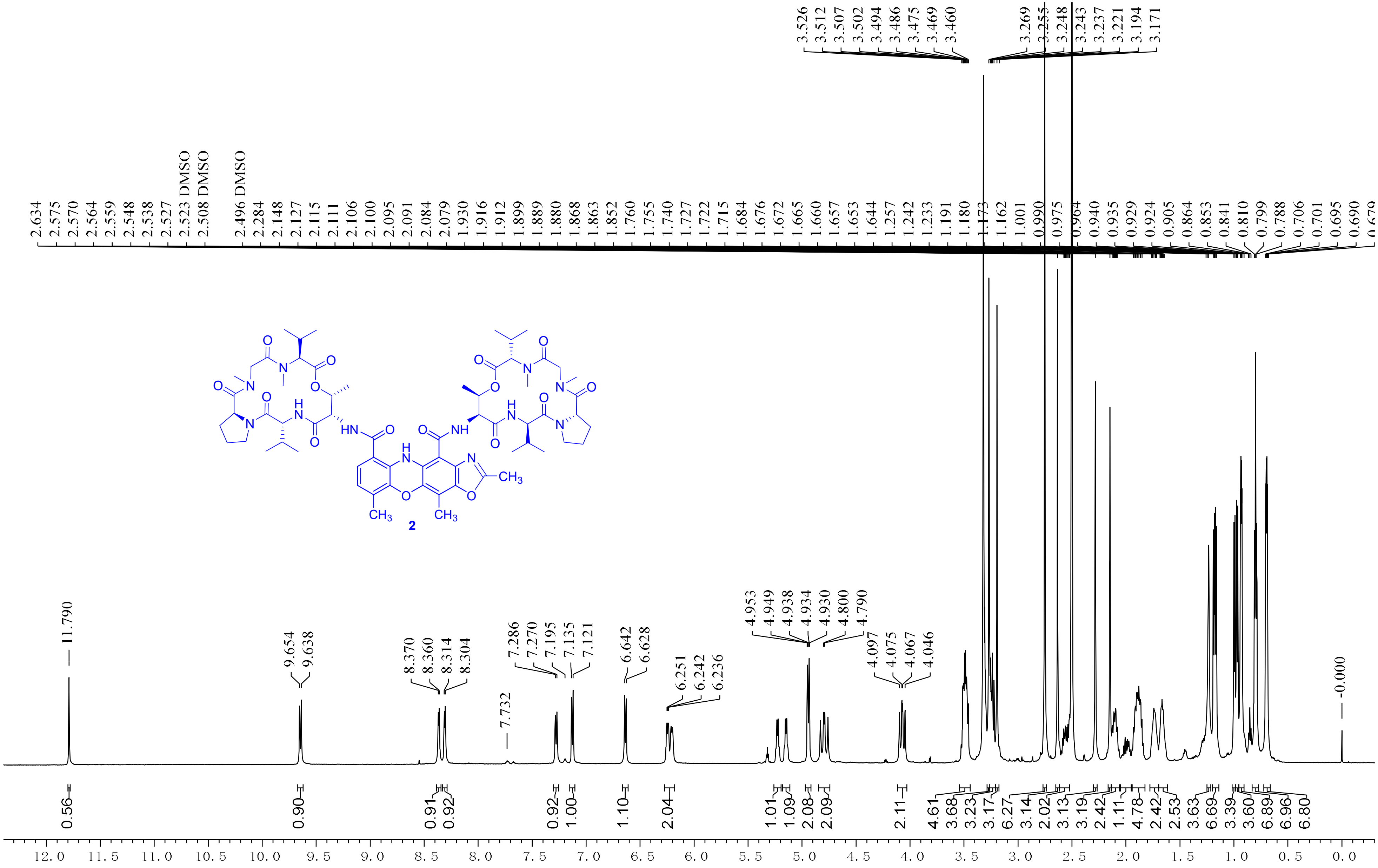

Figure S19. The ^{1}H NMR spectrum of neo-actinomycin B (**2**) in DMSO-d_6 (600 MHz).

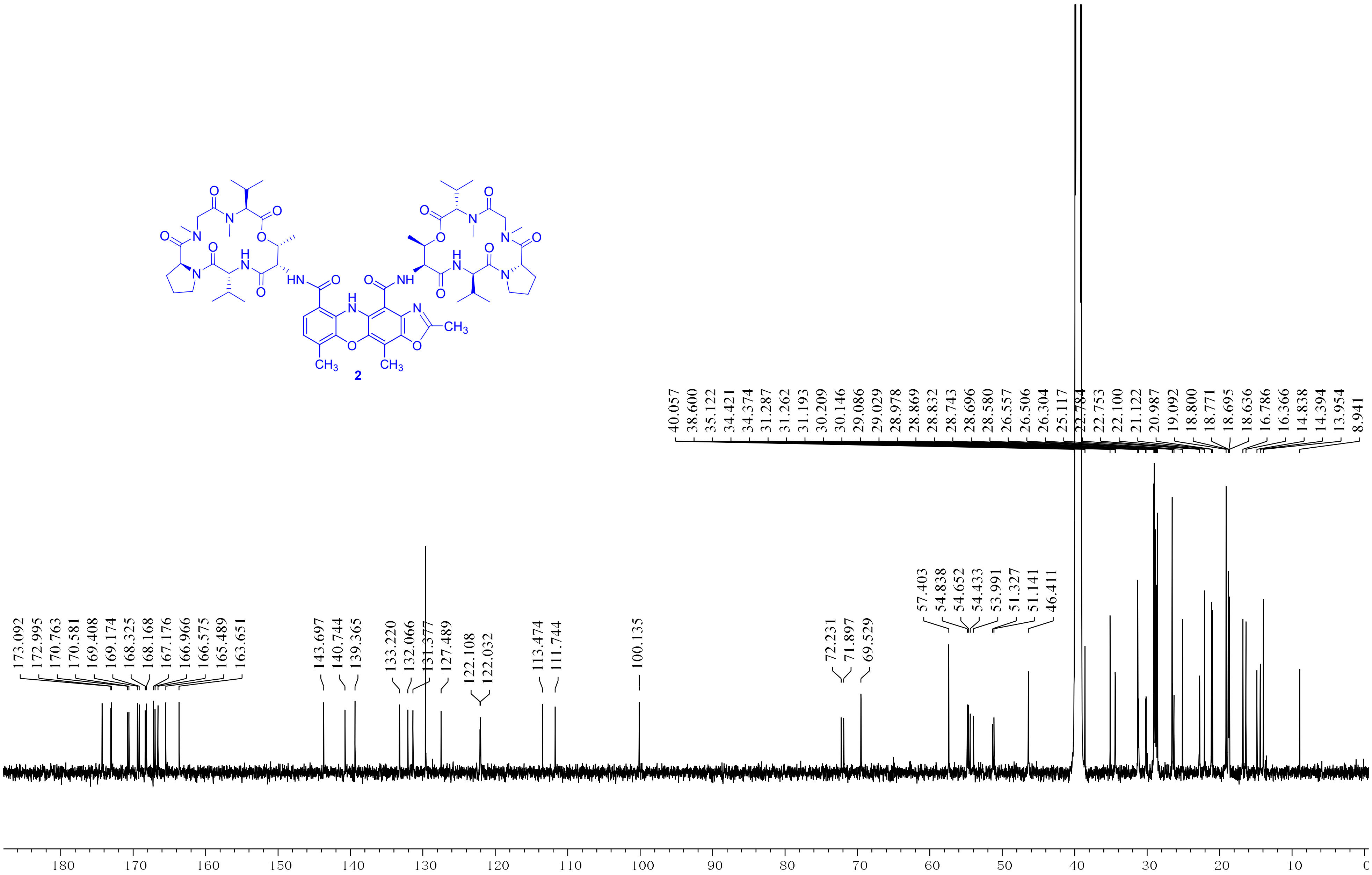

Figure S20. The ^{13}C NMR spectrum of neo-actinomycin B (**2**) in DMSO-d_6 (150 MHz).

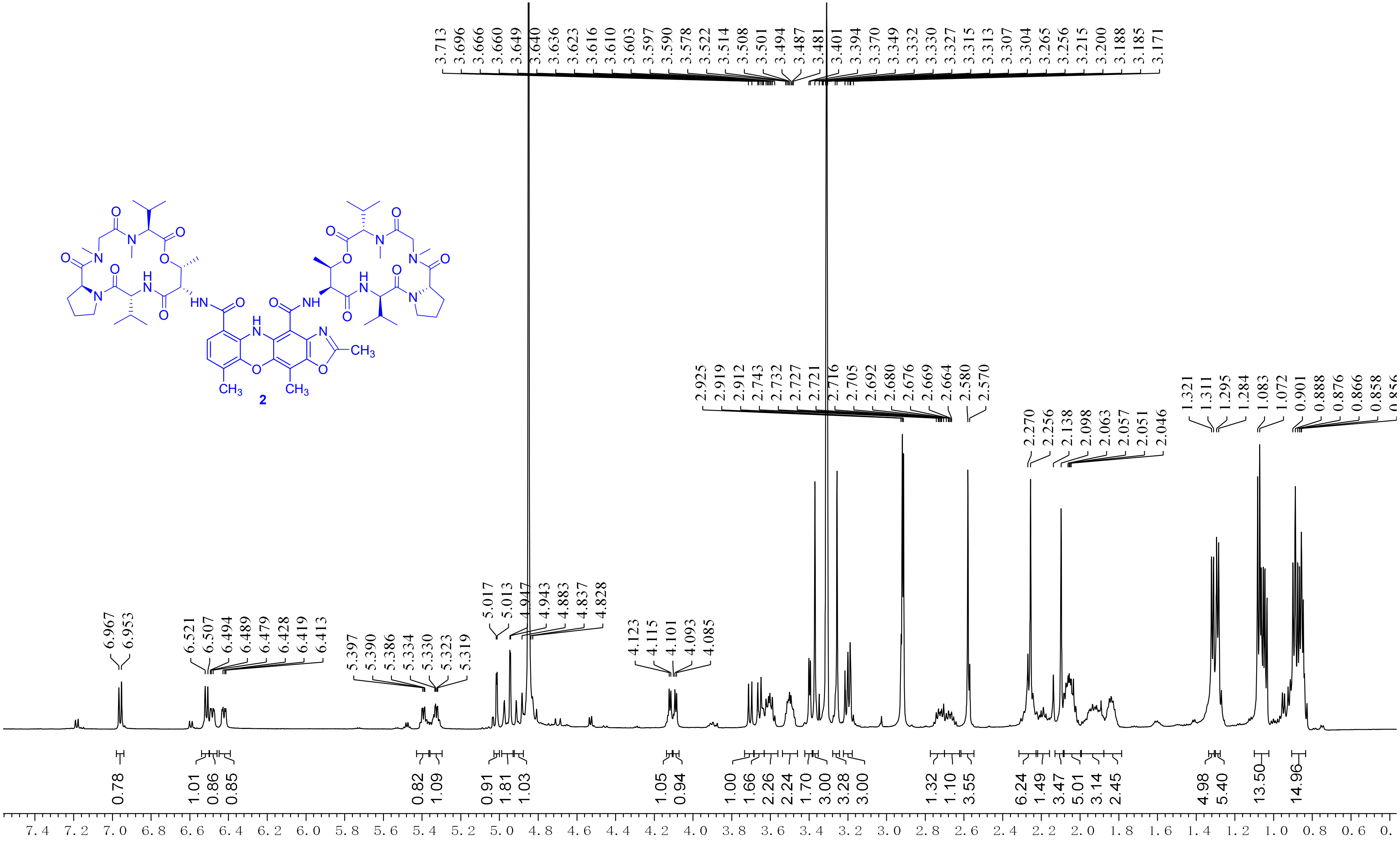

Figure S21. The ^{1}H NMR spectrum of neo-actinomycin B (**2**) in MeOH-d_4 (600 MHz).

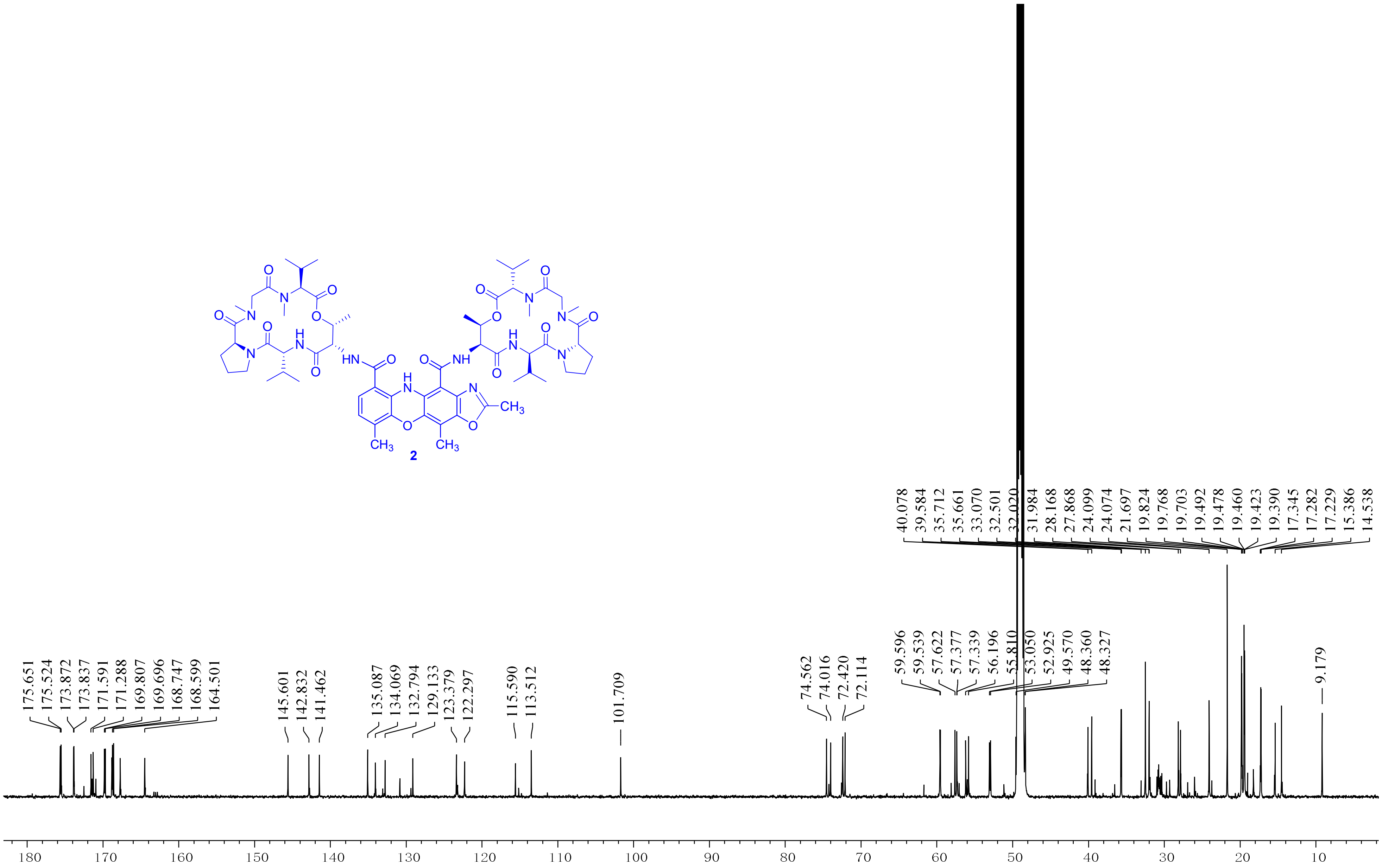

Figure S22. The ^{13}C NMR spectrum of neo-actinomycin B (**2**) in MeOH-d_4 (150 MHz).

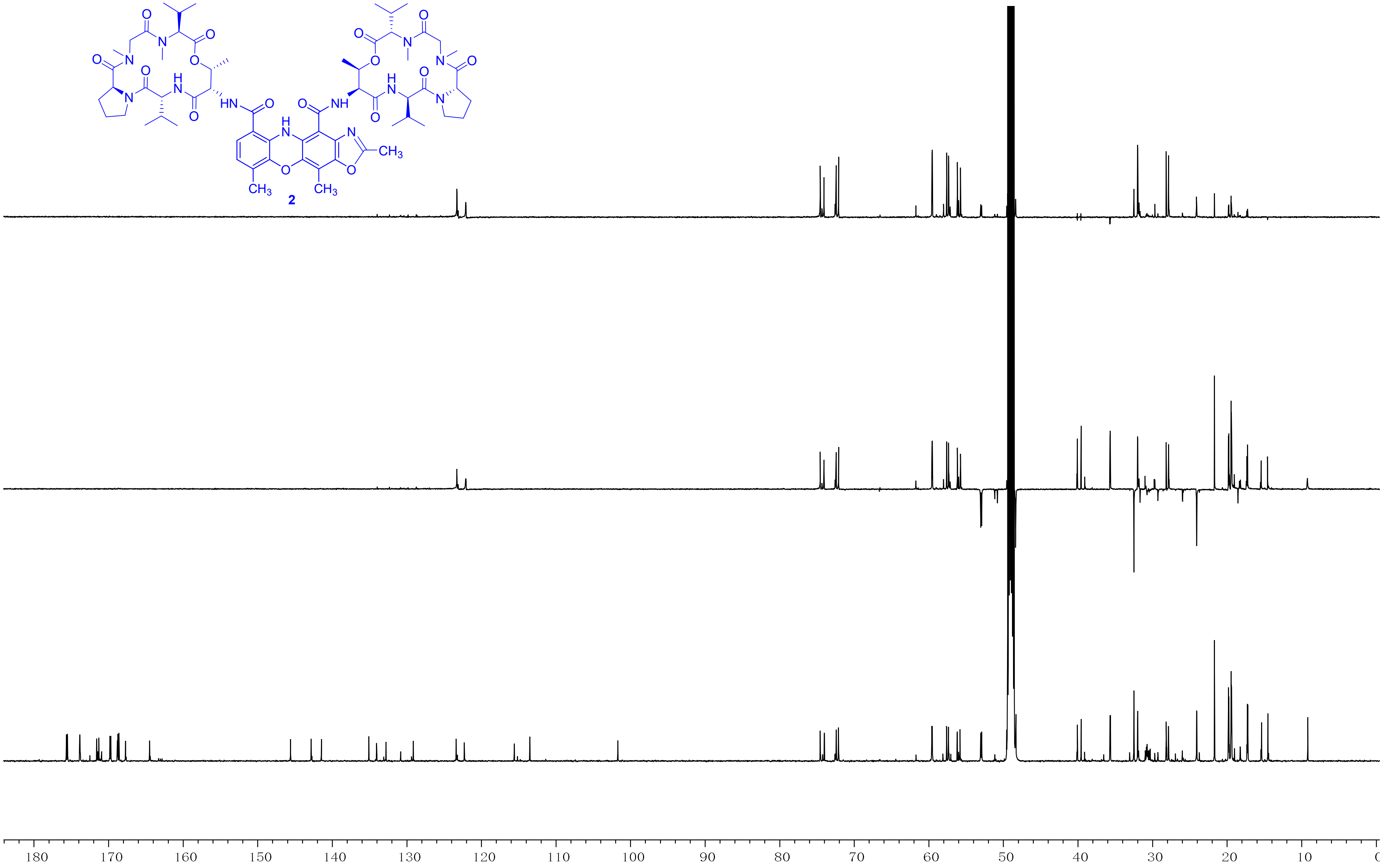

Figure S23. The DEPT spectrum of neo-actinomycin B (**2**) in MeOH-d_4 (150 MHz).

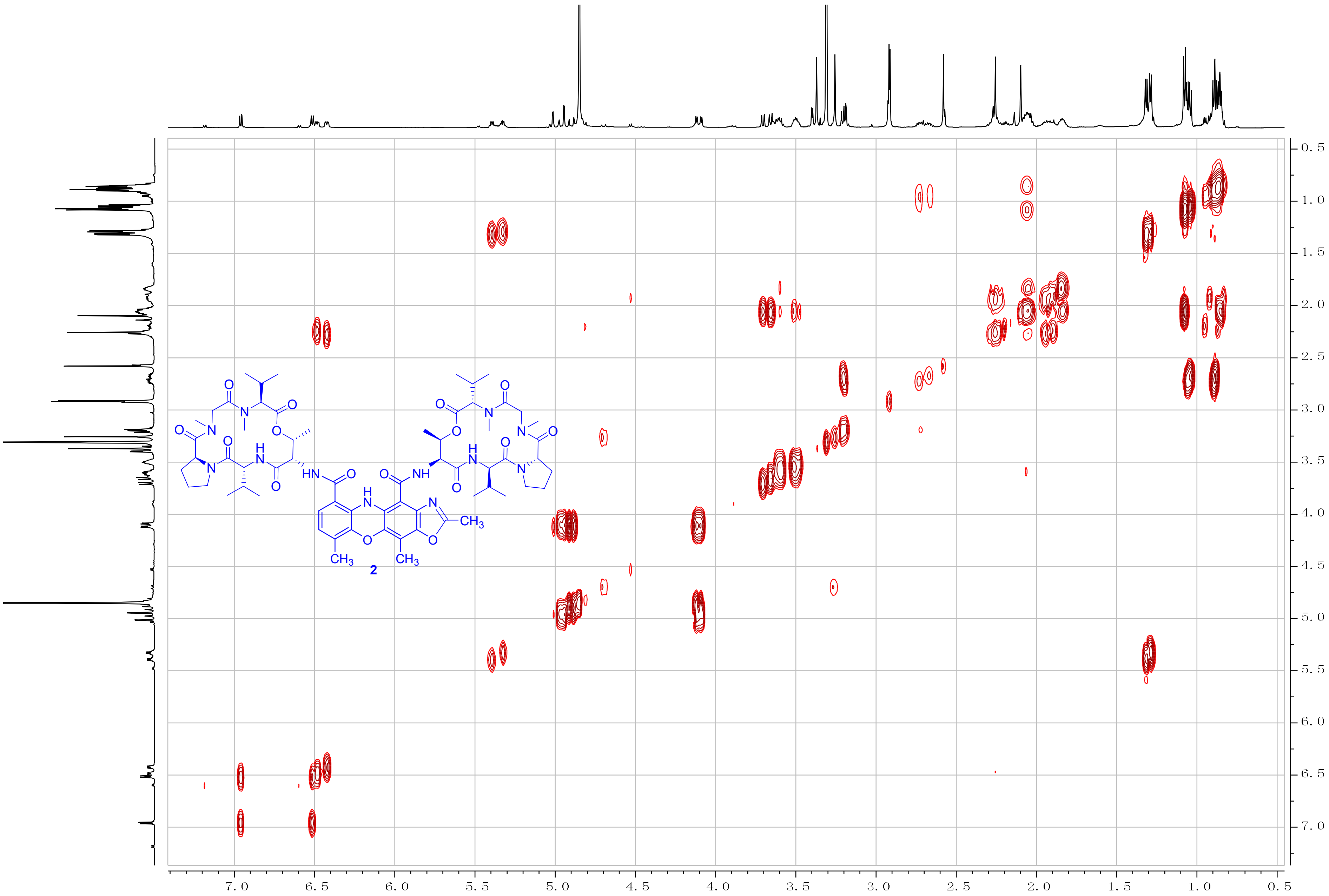

Figure S24. The COSY spectrum of neo-actinomycin B (**2**) in MeOH-d_4 (600 MHz).

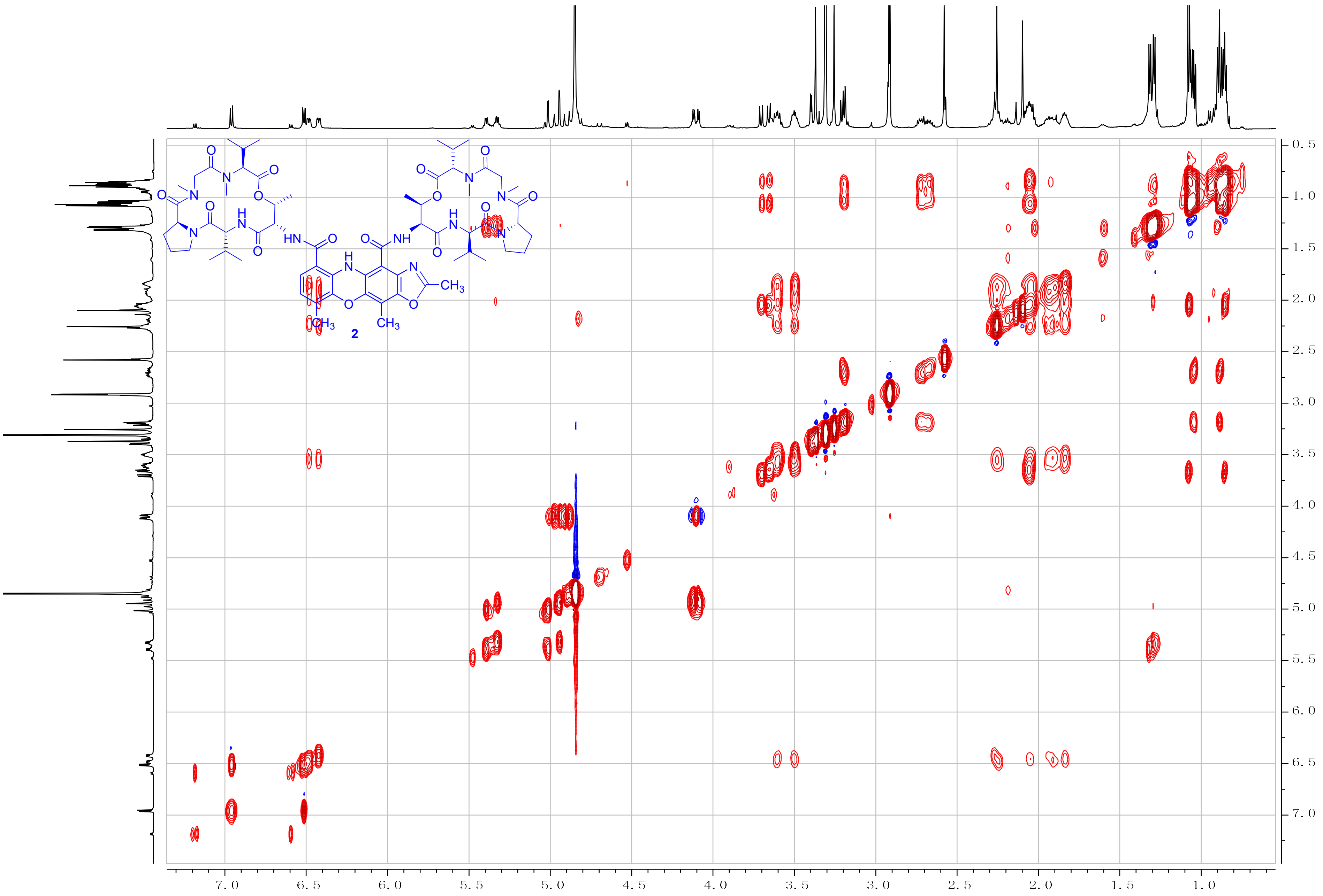

Figure S25. The TOCSY spectrum of neo-actinomycin B (**2**) in MeOH-d_4 (600 MHz).

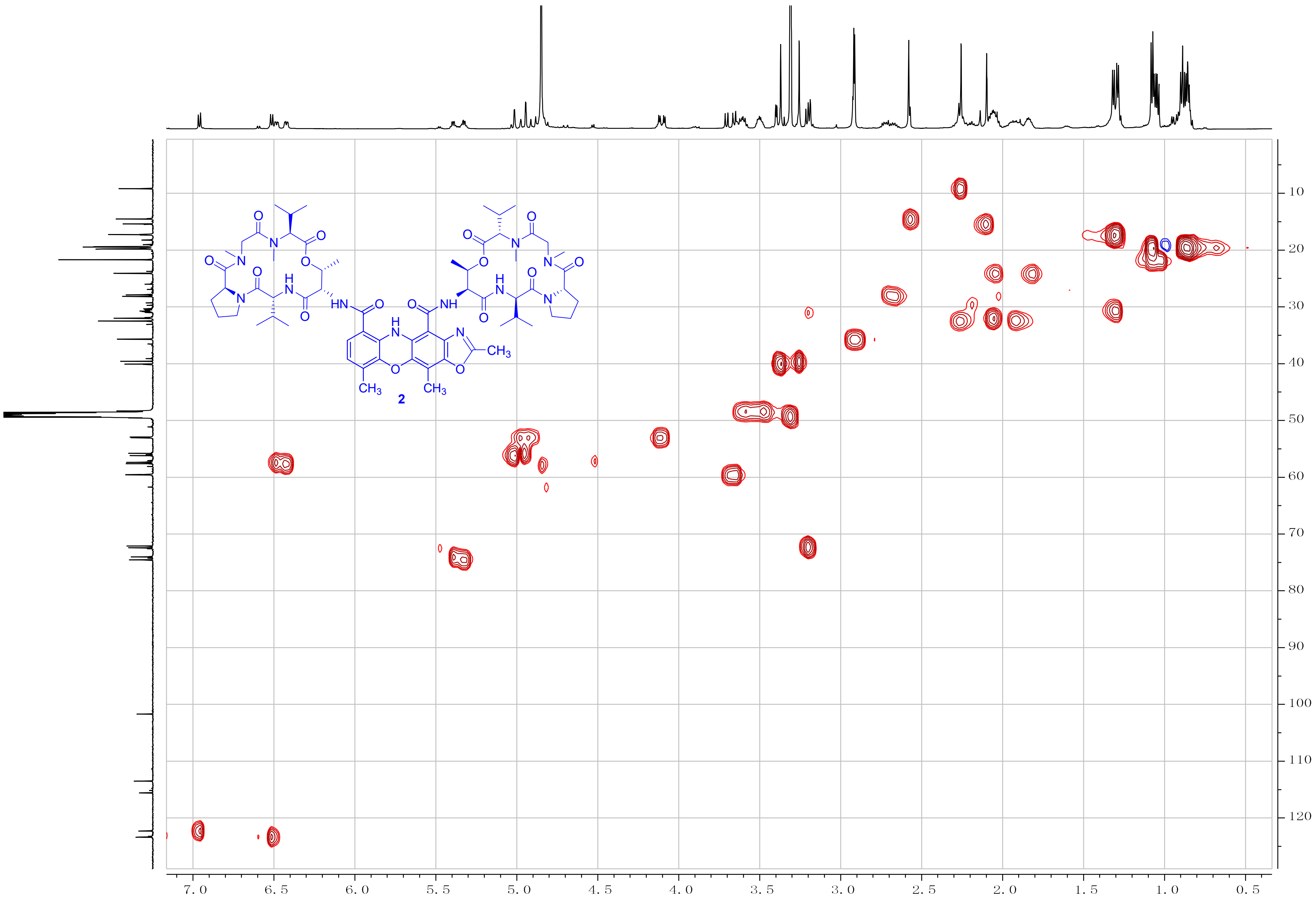

Figure S26. The HSQC spectrum of neo-actinomycin B (**2**) in MeOH-d_4 (600 MHz).

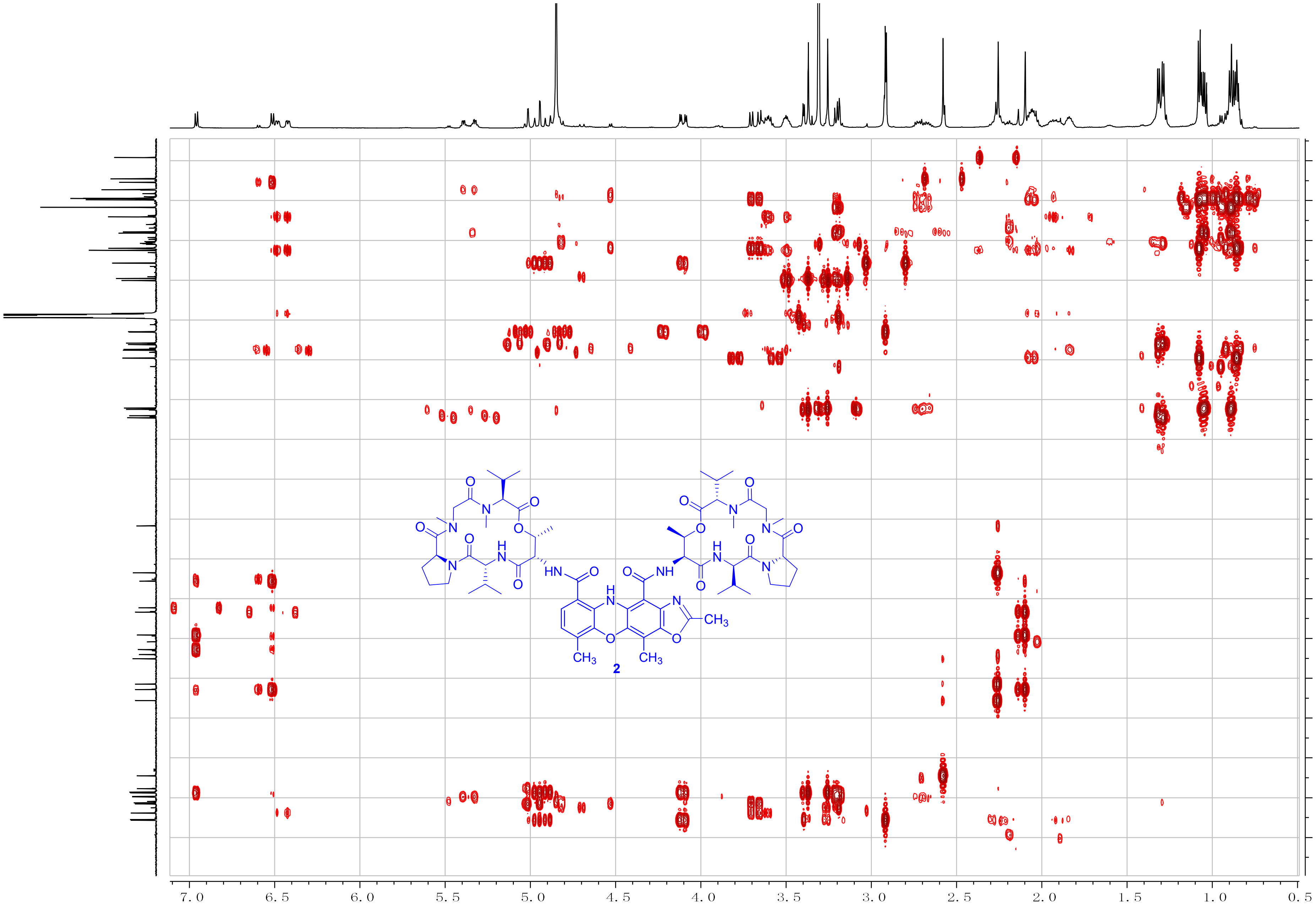

Figure S27. The HMBC spectrum of neo-actinomycin B (**2**) in MeOH-d_4 (600 MHz).

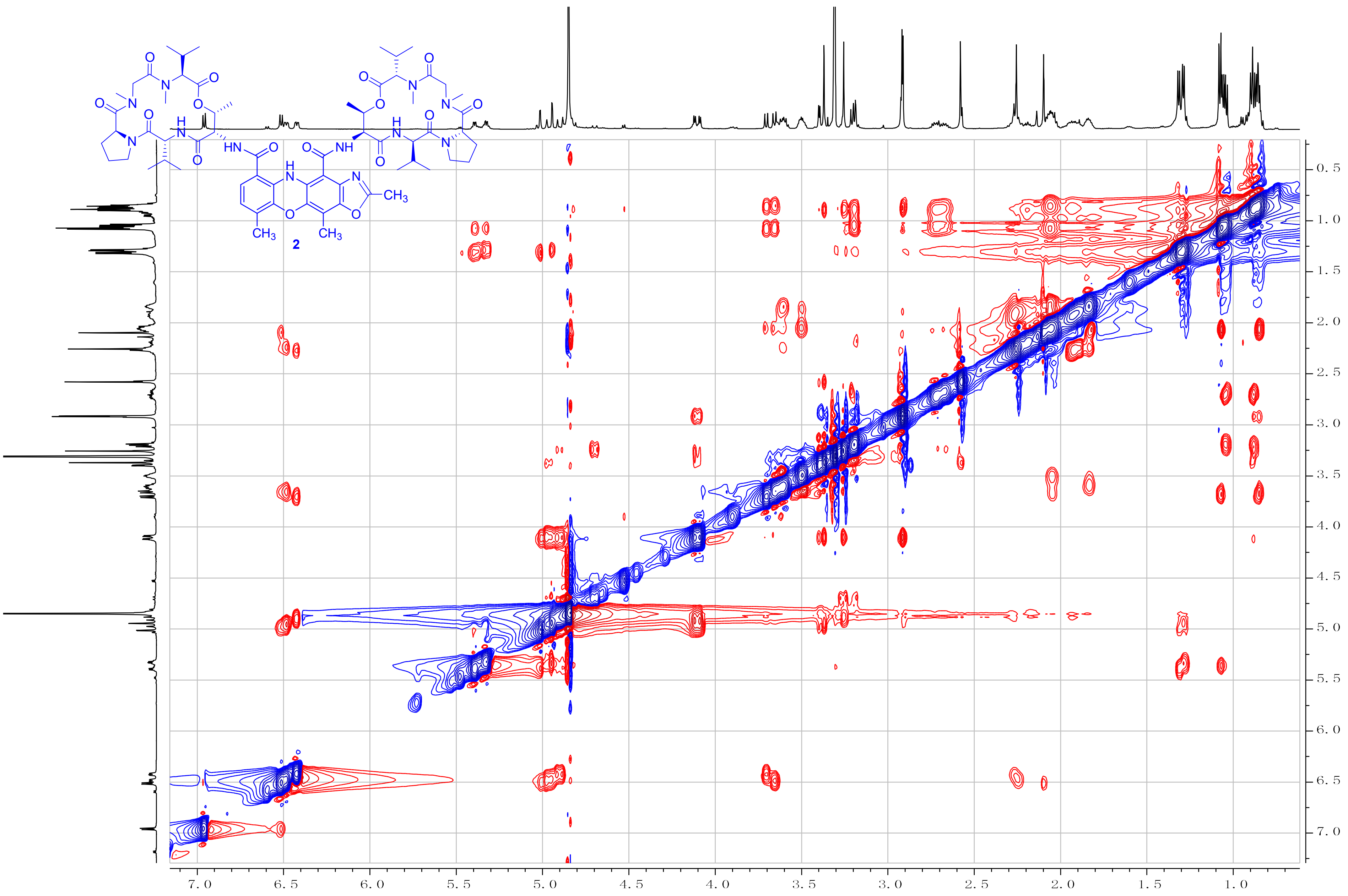

Figure S28. The ROESY spectrum of neo-actinomycin B (**2**) in MeOH-d_4 (600 MHz).
